# Galectin‐1 Elicits a Tissue‐Specific Anti‐Inflammatory and Anti‐Degradative Effect Upon LPS‐Induced Response in an Ex Vivo Model of Human Fetal Membranes Modeling an Intraamniotic Inflammation

**DOI:** 10.1111/aji.70016

**Published:** 2024-11-22

**Authors:** Jazmin Hernández‐Rodríguez, Jesús Pérez‐Hernández, Pilar Flores‐Espinosa, Andrea Olmos‐Ortiz, Pilar Velazquez, Rodrigo Zamora‐Escudero, Marcela Islas‐López, Addy Cecilia Helguera‐Repetto, Karla Hernández‐Bones, Samara Rodríguez‐Flores, Rodrigo Jiménez‐Escutia, Amaury Fortanel‐Fonseca, Arturo Flores‐Pliego, Rosario Lopez‐Vancell, Veronica Zaga‐Clavellina

**Affiliations:** ^1^ Departamento de Inmunobioquímica Instituto Nacional de Perinatología Ciudad de México Mexico; ^2^ Laboratorio de Patología Experimental UME Unidad de Medicina Experimental Facultad de Medicina UNAM Ciudad Universitaria Ciudad de México Mexico; ^3^ Departamento de Ginecología y Obstetricia Hospital Ángeles México Ciudad de México Mexico; ^4^ Ginecología y Obstetricia Hospital Ángeles Lomas‐UNAM Huixquilucan Mexico; ^5^ Posgrado en Ciencias Médicas Escuela Superior de Medicina Instituto Politécnico Nacional Ciudad de México Mexico; ^6^ Posgrado en Ciencias Biológicas Unidad de Posgrado Universidad Nacional Autónoma de México Ciudad de México Mexico; ^7^ Coordinación Áreas Quirúrgicas Hospital Ángeles México Ciudad de México Mexico

**Keywords:** galectin‐1, inflammation, human chorioamniotic membranes, intraamniotic infection, maternal‐fetal unit, preterm birth, preterm labor, tolerogenic

## Abstract

**Problem:**

Intrauterine infection is one of the most jeopardizing conditions associated with adverse outcomes, including preterm birth; however, multiple tolerance mechanisms operate at the maternal–fetal interface to avoid the rejection of the fetus. Among the factors that maintain the uterus as an immunoprivileged site, Galectin‐1 (Gal‐1), an immunomodulatory glycan‐binding protein secreted by the maternal‐fetal unit, is pivotal in promoting immune cell homeostasis. This work aimed to evaluate the role of Gal‐1 during a lipopolysaccharide (LPS)‐induced‐inflammatory milieu.

**Method of Study:**

Using an ex vivo culture with two independent compartments, human fetal membranes at term were pretreated with 40 and 80 ng/mL of Gal‐1, then to reproduce an intraamniotic inflammation, the fetal side of membranes was stimulated with 500 ng/mL of LPS for 24 h. The concentrations of tumor necrosis factor (TNF)‐α, interleukin (IL)‐1β, IL‐6, monocyte chemoattractant protein (MCP1), macrophage inflammatory protein (MIP1) α, regulated upon activation normal T cell expressed and secreted (RANTES), and matrix metalloproteinase (MMP)‐9 were measured in both amnion and choriodecidua compartments.

**Results:**

In a tissue‐specific fashion profile, pretreatment with the physiologic concentration of Gal‐1 significantly diminished the LPS‐dependent secretion of TNF‐α, IL‐1β, Il‐6, MCP1, MIP1α, RANTES, and MMP‐9.

**Conclusion:**

Gal‐1 elicits an anti‐inflammatory effect on the human fetal membranes stimulated with LPS, which supports the hypothesis that Gal‐1 is part of the immunomodulatory mechanisms intended to stop the harmful effect of inflammation of the maternal–fetal interface.

## 1 Introduction

Preterm birth (PTB), considered as parturition occurring before the 37 weeks of gestation, represents a central public health condition that is the primary causal factor of neonatal mortality and morbidity, often with long‐term adverse effects on the health of both mother and child. The World Health Organization states that PTB affects approximately 11% of all live births, representing about 13.4 million PTB annually [1, 2]. In Mexico, 200000 kids born prematurely every year [3].

During pregnancy, intrauterine infection represents a primary endangered condition that compromises the tolerogenic mechanisms capable of maintaining the immune privilege of the maternal‐fetal unit and increases the risk for PTB and preterm premature rupture of membranes (PPROM), which represents a substantial obstetric problem that affects 3%–4% of all pregnancies and precedes 40%–50% of all PTB [4, 5].

In adverse scenarios such as infection and inflammation, the immune competencies of the human fetal membranes represent an essential physical and immunological barricade to block the ascending way of pathogens coming from the lower genital tract [6, 7]. There is robust evidence suggesting that the maternal–fetal interface has multiple pro‐tolerogenic and compensatory mechanisms intending to, at least partially, control inflammation, which can represent a significant risk to the continuity of gestation [8, 9, 10].

Galectins (Gal), a family of glycan‐binding proteins, preferentially recognize various carbohydrates attached to proteins and lipids. These evolutionarily conserved animal lectins can recognize multiple b‐galactose through a conserved carbohydrate‐recognition domain on cell surface glycoconjugates [11]. They are critical in the interactions of cell–cell and cell–extracellular matrix, having essential roles in numerous modulatory functions during pregnancy, including implantation and pregnancy maintenance [12], embryogenesis [13], trophoblast cell function, and placental development of immunological tolerance and angiogenesis [14, 15].

Gal‐1, ‐2, ‐3, ‐4, ‐7, ‐8, ‐9, ‐13, ‐14, ‐15, and ‐16 are substantially expressed in the human maternal–fetal interface [10]. Gal‐1 is a homodimeric protein consisting of two identical subunits from 14.5 KDa highly involved in immuno‐modulatory functions that govern different biological processes requiring tolerance mechanisms in different immune‐privileged sites, including testis, retina, ovary, and placenta [16, 17, 18]. Gal‐1 recognizes galactose‐b1‐4‐Nacetyl‐glucosamine units on the branches of N‐ or O‐linked glycans on diverse cell surfaces [11].

Evidence suggests that Gal‐1 might be “incorporated” by the placenta, fetal membranes, and uterine mucosa during mammalian evolution to confer tolerance through hormonal and redox status regulation [19, 20, 21]. During pregnancy, Gal‐1 is widely expressed at both the mRNA and protein levels in human tissue, being most abundant in decidua and placenta [21, 22, 23, 24, 25, 26].

Clinical evidence indicates that Gal‐1 is upregulated and expressed in human preimplantation embryos during normal pregnancy [27]. Gal‐1 exhibits immunosuppressive properties at the maternal interface in early human pregnancy by modulating HLA‐G expression on trophoblast cells [28]. Additionally, Gal‐1 promotes the generation of tolerogenic dendritic cells (DC), inducing interleukin (IL‐10) expressing T regulatory cells and a Th2 cytokines shift by provoking apoptosis of activated Th1 cells, favoring the establishment of an early tolerogenic milieu during the early stages of gestation [20, 29].

The immunomodulatory role of Gal‐1, maintaining the uterus as an immune‐tolerant tissue, indicates that Gal‐1 secreted by decidual NK cells and other decidual cells contributes to generating an immune‐privileged environment at the maternal–fetal interface. Experimental evidence suggests that Gal‐1 secreted by decidual NKs induces the apoptosis of activated T cells, which can be harmful for gestation [29].

Human fetal membranes express Gal‐7, ‐9, ‐13, ‐14‐, ‐16, ‐17, and Gal‐1, which play pleiotropic intra‐ and extracellular functions. Active forms of Gal‐1 have been immunolocalized in all cells conforming fetal membranes, including amniotic epithelial cells, chorionic trophoblasts, and chorio‐vascular mesenchymal cells [30]. The expression of this protein has been proposed as a key part of the repertory of immunomodulatory mechanisms required by the human fetal membranes to maintain the amniotic cavity as a space immunologically comfortable for the fetus [31].

Key evidence supporting the immunotolerogenic role of Gal‐1 during pregnancy indicates that in comparison with wild type, Gal‐1‐deficient (Lgals1^−/−^) [20] mice show higher rates of fetal loss, and the treatment with Gal‐1 recombinant prevents fetal loss and restored tolerance through multiple mechanisms, including induction of tolerogenic dendritic cells and expansion of IL‐10‐secreting regulatory CD4+ CD25+ T reg cells which usually expand during pregnancy and suppress the maternal allogeneic response directed against the fetus [32, 33]. Additionally, Gal‐1 increases the dendritic cells migration through the extracellular matrix, suggesting a pivotal role in initiating an immune response [16].

Clinical evidence supports the role of Gal‐1 in different pathological scenarios. Proteomic analysis shows that Gal‐1 is upregulated in preterm choriodecidua during PTB, indicating a probable association with the underlying pathology [34]. The immunomodulatory role of Gal‐1 is also supported by the evidence that chorioamnionitis is associated with increased Gal‐1 mRNA expression and strong immunoreactivity of fetal membranes; thus, Gal‐1 may be involved in regulating the inflammatory responses to chorioamniotic infection [35].

On the other hand, the evidence about the role of Gal‐1 in pregnancies complicated with PPROM is contradictory. The Boron DG group recently did not identify significant differences in Gal‐1 and Gal‐9 expression between healthy control placentas and placentas affected by PPROM [36]. However, another study supports that, compared with healthy pregnant women, low Gal‐1 maternal serum level has been associated with the incidence of PPROM [27]; however, there is also information suggesting that maternal serum Gal‐1 and Gal‐3 levels are significantly higher in pregnancies complicated with PPROM, which was interpreted as a possible regulatory effect in key biological processes may be an initiating factor in the pathophysiology of PPROM, a marker in its prediction, and a target of PPROM prevention strategies [27, 37].

The immunomodulatory role of Gal‐1 is also supported by the evidence that chorioamnionitis is associated with increased Gal‐1 mRNA expression and strong immunoreactivity of fetal membranes; thus, Gal‐1 may be involved in regulating the inflammatory responses to chorioamniotic infection [35].

Additionally, a diminution of protein levels of Gal‐1 and Gal‐3 in endometrial tissue was proposed as a cause of endometrial receptivity and also of unexplained infertility [38]. Compared with women with normal singleton pregnancies, Gal‐1 exhibits low expression in serum and placenta of pregnant women with fetal growth restriction, which supposes a role of Gal‐1 in this pathology and could represent a new diagnostic marker of the disease [39].

These data prompted us to investigate the role of Gal‐1 on the in vitro secretion of inflammatory and pro‐degradative markers by human fetal membranes stimulated with lipopolysaccharide (LPS).

## 2 Materials and Methods

### 2.1 Ethic Statement

This study was approved by the Biosafety, Ethical, and Research Committees from the Instituto Nacional de Perinatología (INPer) and is registered under code number INPer 2019‐1‐5. Hospital Angeles‐Mexico (HAM) committees also registered and accepted this protocol. All methodological approaches were conducted according to the Belmont Report and the Declaration of Helsinki; written informed consent was obtained voluntarily from each mother before the cesarean section.

### 2.2 Biological Samples

Biological samples were collected from pregnant women, the normal‐evolutive, uncomplicated term (37–39 weeks), without clinical signals of active labor, who attended their cesarean section in INPer or the HAM and gave their voluntary consent in the presence of two witnesses. Exclusion criteria included: vaginal delivery, cervicovaginal infection during the third trimester, diabetes mellitus, hypertension, obesity, or other metabolic diseases. All patients included in this study lived in Mexico City. They were Hispanic and of middle socioeconomic status. Clinical data from mothers and newborns are described in Table 1. We processed a total number of six chorioamniotic membranes

**TABLE 1:** Clinical parameters of patients who donated fetal membranes.

Clinical parameter	Mean ± SD	Range
	(*n* = 6)	(Min–Max)
Maternal age (years)	31.5 ± 3.44	(28–37)
Gestacional age (weeks)	38.15 ± 1.06	(37–40)
Number of pregnancies	1.66 ± 0.81	(1–3)
Newborn cephalic perimeter (cm)	33.25 ± 1.25	(32–35)
Newborn length (cm)	48.83 ± 0.98	(47–50)
Newborn weigth (g)	3159.33 ± 403.13	(2675–3625)
Apgar	9 ± 0	(9–9)
Newborn sex (male/female) (%/%)	2/4	(33.3%/66.6%)

### 2.3 Fetal Membrane Culture

In the present study, we used an experimental model initially validated and published by our group. The fully functional fetal membranes are cultured in a two‐compartment system that maintains viability and responsiveness of the whole membrane and in paracrine communication [40, 41].

Briefly, fetal membranes were manipulated under sterile conditions and were rinsed in a sterile 0.9% NaCl solution to remove blood clots, and the whole membrane was visually inspected to ensure mechanical integrity. Fetal membranes were manually cut with a scalpel into ∼3 × 3 cm and were placed over a synthetic membrane‐depleted transwell device (Costar, New York, NY, USA). The explants were held in place using sterile silicone rubber rings. The choriodecidual side was oriented in the upper chamber of the transwell, whereas the amniotic side was oriented lower. This model allowed us to evaluate the amnion and choriodecidua secretions independently [41]. The two independent compartment models were placed into a 12‐well culture dish and maintained for 24 h in DMEM (Dulbecco's modified Eagle's medium)‐supplemented culture medium (DMEM + 10% FBS + 1 mM sodium pyruvate + penicillin 100 U/mL, streptomycin 100 µg/mL, and amphotericin B 25 ng/mL) in each side of the chamber in a humidified incubator at 37°C and 5% CO_2_–95% air. Additionally, small tissue sections were maintained for 3 days in selective bacterial media culture to ensure the absence of subclinical chorioamniotic infection by *Escherichia coli*, *Staphylococcus aureus*, *Streptococcus agalactiae*, *Candida albicans*, *Gardnerella vaginalis*, *Neisseria gonorrhoeae*, *Lactobacillus* sp., *Klebsiella* sp., *Ureaplasma urealyticum*, or *Mycoplasma hominis*.

### 2.4 Stimulation of Human Fetal Membranes

After 24 h in culture required for adaptation, the culture medium was restored with fresh DMEM + 10% lactalbumin hydrolyzate + 1 mM sodium pyruvate + penicillin 100 U/mL, streptomycin 100 µg/mL, and amphotericin B 25 ng/mL in each side of the chamber in a humidified incubator at 37°C and 5% CO_2_–95% air. Simultaneously, amnion and choriodecidua were stimulated with two physiologic concentrations [28] of 40 or 80 ng/mL of Gal‐1 (human recombinant Pepro Tech, NJ, USA) for 24 h, then the medium and the stimulus with Gal‐1 were renewed, and at this moment, with the intention to emulate an intraamniotic inflammation, the amnion was stimulated with 500 ng/mL of LPS. The co‐stimulation with Gal‐1 and LPS was done for 24 h (in triplicate), then the culture medium was frozen and stored at −70°C until cytokine quantification. Controls were included with lactose (Lac) 30 mM as an inhibitor of Gal‐1 activity [42, 43] and Dexamethasone (Dxm) 200 nM as a positive anti‐inflammatory control.

The explants were immediately processed with protein lysis buffer (20 mM Tris HCl, 150 mM NaCl, 1 mM MgCl_2_, 1 mM EGTA supplemented with 1:1000 P8340 protease inhibitor [Sigma‐Aldrich, St Louis, MO, USA]), disrupted by vortex agitation for 10 min at 4°C, and sonicated at 80 W in 30 s cycles at 4°C for 15 min in an ultrasonic bath sonicator (Bioruptor Pico Sonication Device, Diagenode, Denville, NJ, USA). The samples were centrifuged at 3500 rpm for 15 min at 4°C, and the supernatant was collected and stored at −70°C until the cytokines and chemokines were quantified. After thawing, the protein content of the tissue lysates was quantified using the Bradford method. The tissue lysates were used for zymography.

### 2.5 Cytokine and Chemokine Quantification by ELISA

An ELISA commercial kit evaluated tumor necrosis factor (TNF‐α) and IL‐1β secretion in the culture medium (DTA00D, R&D Systems, MN, USA, and 900‐K95, Peprotech, NJ, USA, respectively). Both kits had a detection limit of 15 pg/mL. Cytokine levels were normalized per gram of wet tissue.

The concentrations of chemokines monocyte chemoattractant protein (MCP‐1) (900‐K31; Peprotech), macrophage inflammatory protein (MIP)‐1α (900‐K35; Peprotech), and regulated upon activation normal T cell expressed and secreted (RANTES) (900‐K33; Peprotech), present in cell culture supernatants from maternal and fetal compartments were determined by Sandwich ELISA according to the manufacturer's instructions.

### 2.6 Zymography

SDS‐polyacrylamide gels (7.5%) co‐polymerized with porcine gelatin 1% (Sigma) were prepared according to standard protocols. Different protein concentrations were loaded: 12 µg from tissues and 10 µg from culture media. U937 conditioned medium was used as a control for matrix metalloproteinase (MMP)‐9 activity. Electrophoresis was run under non‐denaturing conditions, at constant current (120 V) in ice bed for 2 h. After that, gels were washed in 2.5% Triton 100× for 30 min to eliminate SDS and incubated overnight at 37°C in a buffer with pH 7.4 (Tris Base 50 mM, NaCl 0.15 M, CaCl_2_ 20 mM, and 0.02% NaN_3_). Gels were stained with Coomassie blue R‐250. The images were acquired with the Versadoc (Biorrad) equipment with Quantity one 4.6.3 software. Densitometric analysis was performed using ImageJ 1.53k National Institutes of Health USA software.

### 2.7 Histologic Evaluation of Fetal Membranes

After treatments, fetal membranes were fixed in parafolmadehyde 4% solution and embedded in paraffin wax. The histologic slides were stained with Hematoxylin‐Eosin (Sigma, St Louis, Missouri, USA) and coverslipped. The slides were assessed by light microscopy (Zeiss Axio Lab.A1, Jena, Germany).

### 2.8 Statistics Analysis

The cytokine and chemokine immunoassay results were analyzed through a non‐parametric one‐way analysis of variance (ANOVA) Kruskal–Wallis using Sigma Stat v 11 (Systat Software, SigmaStat Version 13, from Systat Software, Inc.; systatsoftware.com). A *p* value ≤ 0.05 was considered statistically significant.

## 3 Results

### 3.1 Anti‐Inflammatory Role of Gal‐1 in Human Fetal Membranes

To evaluate if maternal serum concentration of Gal‐1 during pregnancy [28] can promote an immunomodulatory response in human fetal membranes, we exposed these tissues to Gal‐1 for 24 h. Then, we stimulated a pro‐inflammatory response with LPS in the amniotic compartment, mimicking an intraamniotic infection. TNF‐α, IL‐6, IL‐1β, MCP‐1, RANTES, MIP‐1α, and IL‐8 compartmentalized secretion by choriodecidua, and amnion were studied.

IL‐1β is a cytokine basally produced by both amnion (155 [123–180] pg/mL/g of tissue) and choriodecidua (722 [425–1315] pg/mL/g of tissue). Intraamniotic stimulus with LPS induced 17.3 times IL‐1β secretion (2693 [1718–3204] pg/mL/g of tissue) in amnion region, and 6 times (4411 [4106–4551] pg/mL/g of tissue) in choriodecidua region. In comparison with LPS treatment, Gal‐1 at concentrations of 40 ng/mL or 80 ng/mL in co‐stimulation with LPS downregulated the IL‐1β secretion in choriodecidua by 60.6% (1737 [1261–2114] pg/mL/g of tissue) and 53.4% (2055 [1027–2274] pg/mL/g of tissue), respectively, whereas diminished by 78.8% (570 [462–774] pg/mL/g of tissue) and 87.55% (335 [257–942] pg/mL/g of tissue) in amnion region. The co‐treatment with Lac and Gal‐1 reverted the anti‐IL‐1β secretion in the choriodecidua (3650 [2893–4372] pg/mL/g of tissue) but not in the amnion region (1155 [626–1930] pg/mL/g of tissue).

**FIGURE 1:**
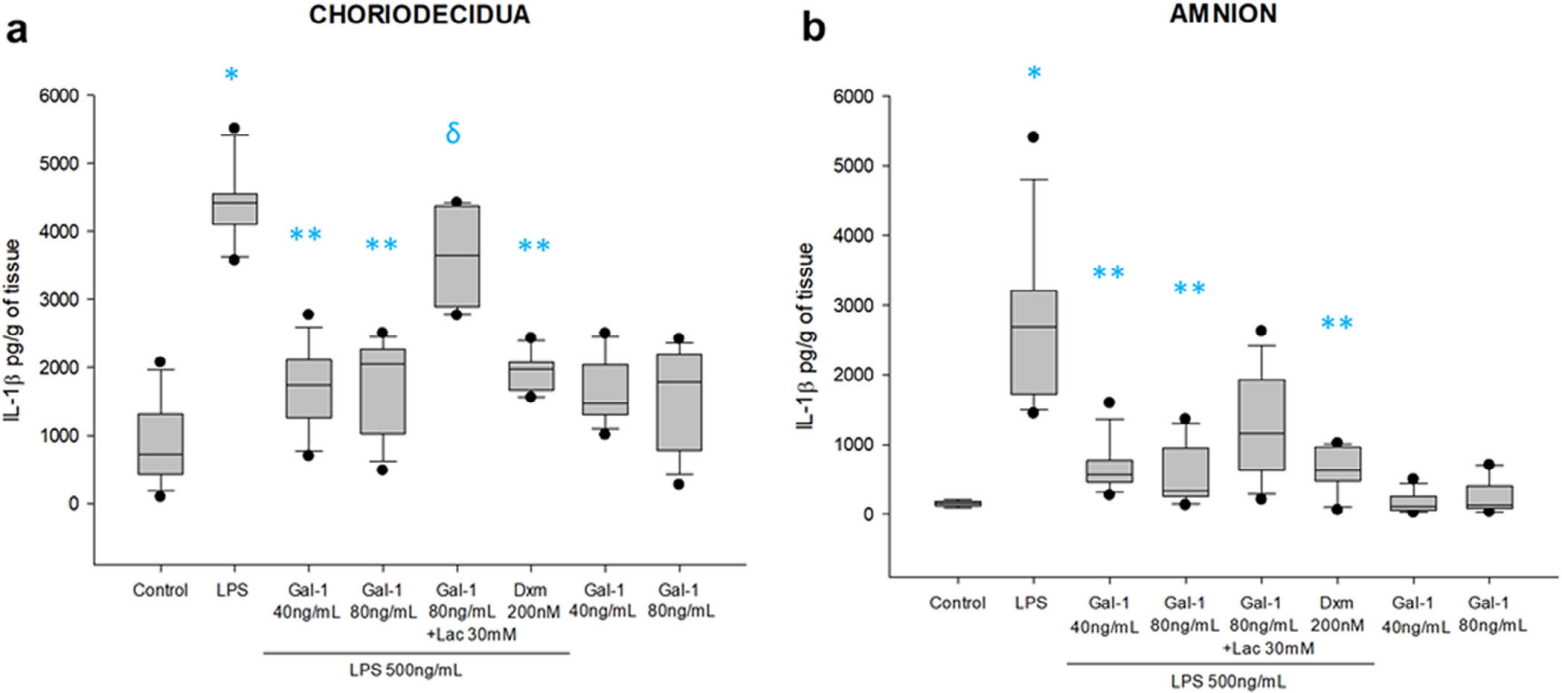
IL‐1β secretion profile in choriodecidua and amnion. Quantification by ELISA (a) in choriodecidua and (b) amnion culture medium. Plots are presented as boxes and whiskers: graphs show medians with interquartile range. Outliers are indicated by closed circles. LPS: lipopolysaccharide 500 ng/mL, Dxm: dexamethasone (200 nM), lac: Lac (30 mM). Significant differences versus * control, **LPS, and ẟ versus Gal‐1 80ng+LPS (*p* ≤ 0.05) are indicated.

The co‐treatment of explants with Gal‐1 and Lac reverted the effect of Gal‐1 in both compartments; however, it was significant only in the choriodecidua region. Compared with LPS alone, the co‐treatment of LPS and Dxm significantly diminished the secretion of IL‐1β in both compartments (Figure 1).

**FIGURE 2:**
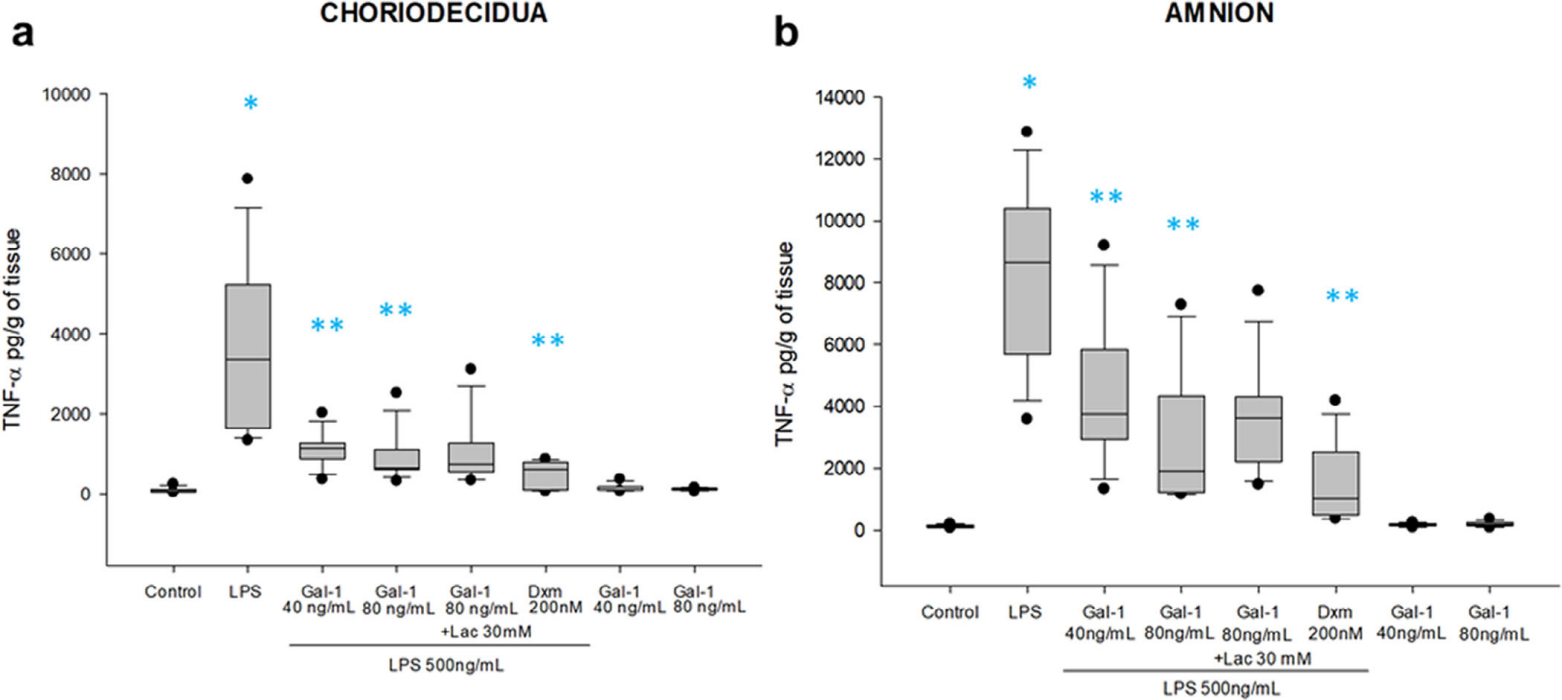
TNF‐α secretion profile in chorion and amnion. Quantification by ELISA (a) in chorion and (b) amnion culture medium. Plots are presented as boxes and whiskers: graphs show medians with interquartile range. Outliers are indicated by closed circles. LPS: lipopolysaccharide 500 ng/mL, Dxm: dexamethasone (200 nM), lac: Lac (30 mM). Significant differences versus * control, **LPS, and ẟ versus Gal‐1 80ng+LPS (*p* ≤ 0.05) are indicated.

In the choriodecidua region, the basal level of TNF‐α (60 [45–108] pg/mL/g of tissue) significantly increased (3361 [1647–5237] pg/mL/g of tissue) 55.6 times after the stimulation with LPS. In comparison with basal level (104 [83–157] pg/mL/g of tissue), the stimulation with LPS in the amnion induced 82.8 times the concentration of TNF‐α (8649 [5862–10 401] pg/mL/g of tissue). Both concentrations of Gal‐1 were effective to promote an anti‐inflammatory effect by diminishing 66% and 81% of this cytokine in the choriodecidual region, and by 56% and 77.8% in the amnion region. In this case, the treatment with Lac could not block the anti‐TNF‐α effect of Gal‐1 (Figure 2).

In both compartments and in comparison, with LPS alone, the co‐treatment with Dxm significantly reduced the production of TNF‐α. Lac partially reverted the effect of Gal‐1, but its effect was not statistically significant (Figure 2).

**FIGURE 3:**
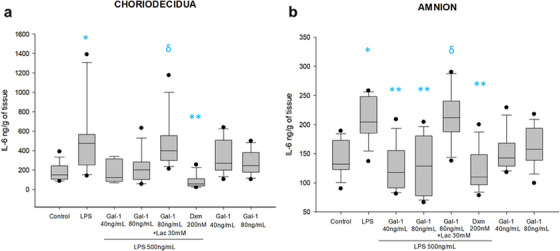
IL‐6 secretion profile in chorion and amnion. Quantification by ELISA (a) in chorion and (b) amnion culture medium. Because the data did not show a normal distribution, they are presented as boxes and whiskers: graphs show medians with interquartile range. Outliers are indicated by closed circles. LPS: lipopolysaccharide 500 ng/mL, Dxm: dexamethasone (200 nM), lac: Lac (30 mM). Significant differences versus * control, **LPS, and ẟ versus Gal‐1 80ng+LPS (*p* < 0.05) are indicated.

IL‐6 is basally secreted by choriodecidual (151 [105–243] ng/mL/g of tissue) and amnion (132 [123–173] ng/mL/g of tissue) regions. IL‐6 secretion rose 3.1 (475 [251–567] ng/mL/g of tissue) and 1.5 (204 [185–248] ng/mL/g of tissue) times, respectively, once the amnion was stimulated with 500 ng/mL of LPS. In comparison with LPS alone, the co‐treatment with 40 and 80 ng/mL of Gal‐1 downregulates the concentration of IL‐6 in a significant pattern, resulting in levels of 117 (91–155) and 128 (77–180) ng/mL/g of amnion, and 122 (81–315) and 202 (103–283) ng/mL/g of choriodecidua. In both compartments, the co‐treatment with Lac 30 mM reverted the anti‐IL‐6 effect of Gal‐1 significantly (Figure 3).

The co‐treatment with Lac reverted the effect of Gal‐1 upon the production of IL‐6 in both amnion and the choriodecidua regions. On the other hand, compared with the control stimulated with only LPS, the control with Dxm and LPS showed a significant decrease in the level of IL‐6 (Figure 3).

### 3.2 Anti‐Chemotactic Role of Gal‐1 in Human Fetal Membranes

**FIGURE 4:**
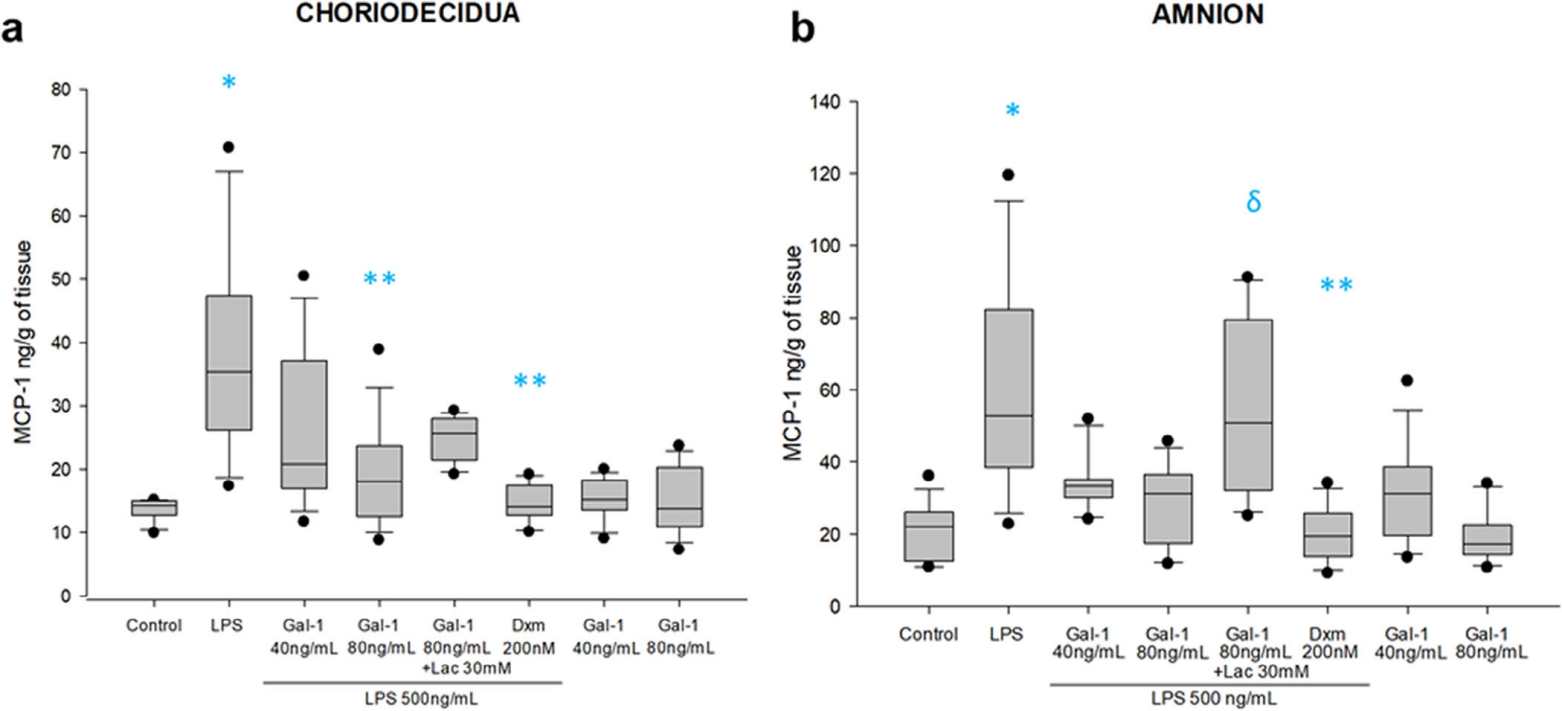
Secretion profile of MCP‐1 in chorion and amnion. Quantification by ELISA in (a) chorion and (b) amnion culture medium. Plots are presented as boxes and whiskers: graphs show medians with interquartile range. Outliers are indicated by closed circles. LPS: lipopolysaccharide 500 ng/mL, Dxm: dexamethasone (200 nM), lac: Lac (30 mM). Significant differences versus * control, **LPS, and ẟ versus Gal‐1 80ng+LPS (*p* ≤ 0.05) are indicated.

In relation to chemokines, choriodecidua and amnion basally secreted MCP‐1 (14.2 [12.7–15] ng/mL/g of tissue and 22.1 [12.4–26] ng/mL/g of tissue, respectively). Amniotic treatment with LPS significantly induced MCP‐1 secretion in both amnion and choriodecidua compartments (53 [38–82] ng/mL/g of tissue and 35 [26–47] ng/mL/g of tissue, respectively). Treatment with Gal‐1 diminished the choridecidual secretion of MCP‐1 in 41% (20 [17–37] ng/mL/g) and 49% (18 [12–23] ng/mL/g) of tissue in comparison with LPS treatment; in turn, Gal‐1 diminished the amniotic secretion of MCP‐1 in 36% (33 [30–35] ng/mL/g) and 40% (31 [17–36] ng/mL/g) of tissue. The co‐treatment with Lac reverted the anti‐MCP‐1 secretion in the amnion but not in the choriodecidual region. The amnion was the only region where the co‐treatment with Lac reverted the inhibitory effect of Gal‐1 on MCP‐1 secretion. On the other hand, as we expected, the Dxm decreased significantly in the level of MCP‐1 in both choriodecidual and amniotic regions (Figure 4).

**FIGURE 5:**
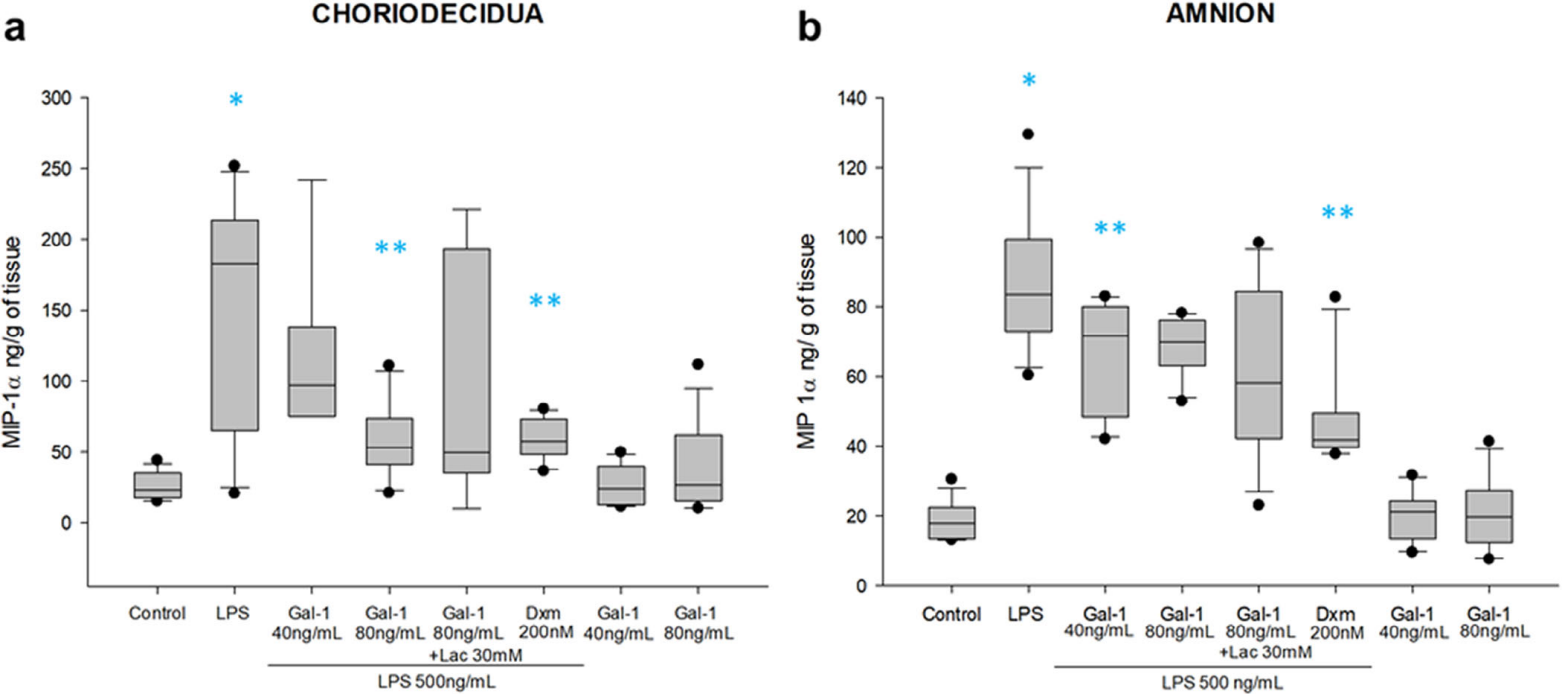
Secretion profile of MIP‐1α in chorion and amnion. Quantification by ELISA in (a) chorion and (b) amnion culture medium. Plots are presented as boxes and whiskers: graphs show medians with interquartile range. Outliers are indicated by closed circles. LPS: lipopolysaccharide 500 ng/mL, Dxm: dexamethasone (200 nM), lac: Lac (30 mM). Significant differences versus * control, **LPS, and ẟ versus Gal‐1 80ng+LPS (*p* ≤ 0.05) are indicated.

LPS stimulation in the amnion region significantly induced MIP‐1α secretion by 7.8 times and 4.6 times in both choriodecidual (183 [65–213] ng/mL/g of tissue) and amnion (83 [73–94] ng/mL/g of tissue) compartments, in comparison to the basal state. Fetal membranes exposed to both doses of Gal‐1 significantly diminished their MIP‐1α secretion in choriodecidual (97 [75–138], and 53 [41–73] ng/mL/g of tissue) and in amnion (71 [48–80], and 70 [63–76] ng/mL/g of tissue), compared with explants treated with LPS alone. The treatment with Lac could not revert the anti‐ MIP‐1α effect of Gal‐1 (Figure 5).

On the other hand, although there is a tendency to show that the co‐treatment with Lac reverted the effect of Gal‐1, the differences were not significant. The anti‐inflammatory control with Dxm reduced the level of MIP‐1α significantly when is compared to LPS alone (Figure 5).

**FIGURE 6:**
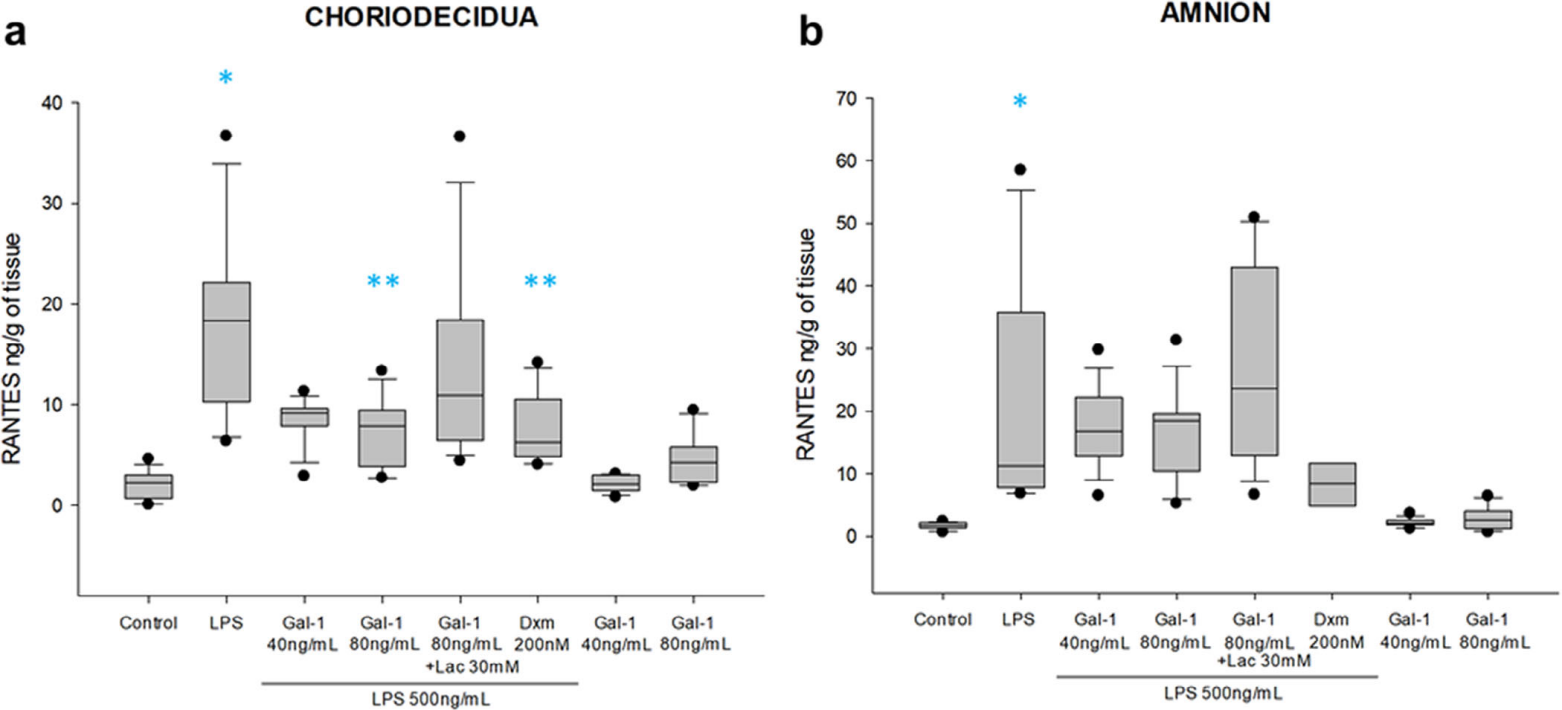
RANTES secretion profile in chorion and amnion. Quantification by ELISA in (a) chorion and (b) amnion culture medium. Plots are presented as boxes and whiskers: graphs show medians with interquartile range. Outliers are indicated by closed circles. LPS: lipopolysaccharide 500 ng/mL, Dxm: dexamethasone (200 nM), lac: Lac (30 mM). Significant differences versus * control, **LPS, and ẟ versus Gal‐1 80ng+LPS (*p* ≤ 0.05) are indicated.

LPS treatment also significantly induced RANTES secretion in choriodecidua (18.3 [10.3–22.1] vs. 2.2 [0.7–3.0] ng/mL/g of tissue) and amnion (11.2 [7.7–35.7] vs. 1.6 [1.3–2.1] ng/mL/g of tissue). Pre‐treatment with both doses of Gal‐1 significantly diminished the RANTES induction by LPS in the choriodecidual compartment (9.1 [7.8–9.6] and 7.8 [3.8 and 9.4] ng/mL/g of tissue); this anti‐chemokine effect of Gal‐1 could not be reverted by Lac treatment (10.8 [6.4–18.3] ng/mL/g of tissue). Any of the concentrations of Gal‐1 downregulated RANTES in the compartment delimited by amnion (Figure 6). The co‐treatment with Lac partially reverted the effect of Gal‐1; however, the increase in the concentration of RANTES was not significant. On the other hand, the control with Dxm decreased the concentration of this RANTES in both regions; nonetheless, it was statistically significant only in the choriodecidua region (Figure 6).

**FIGURE 7:**
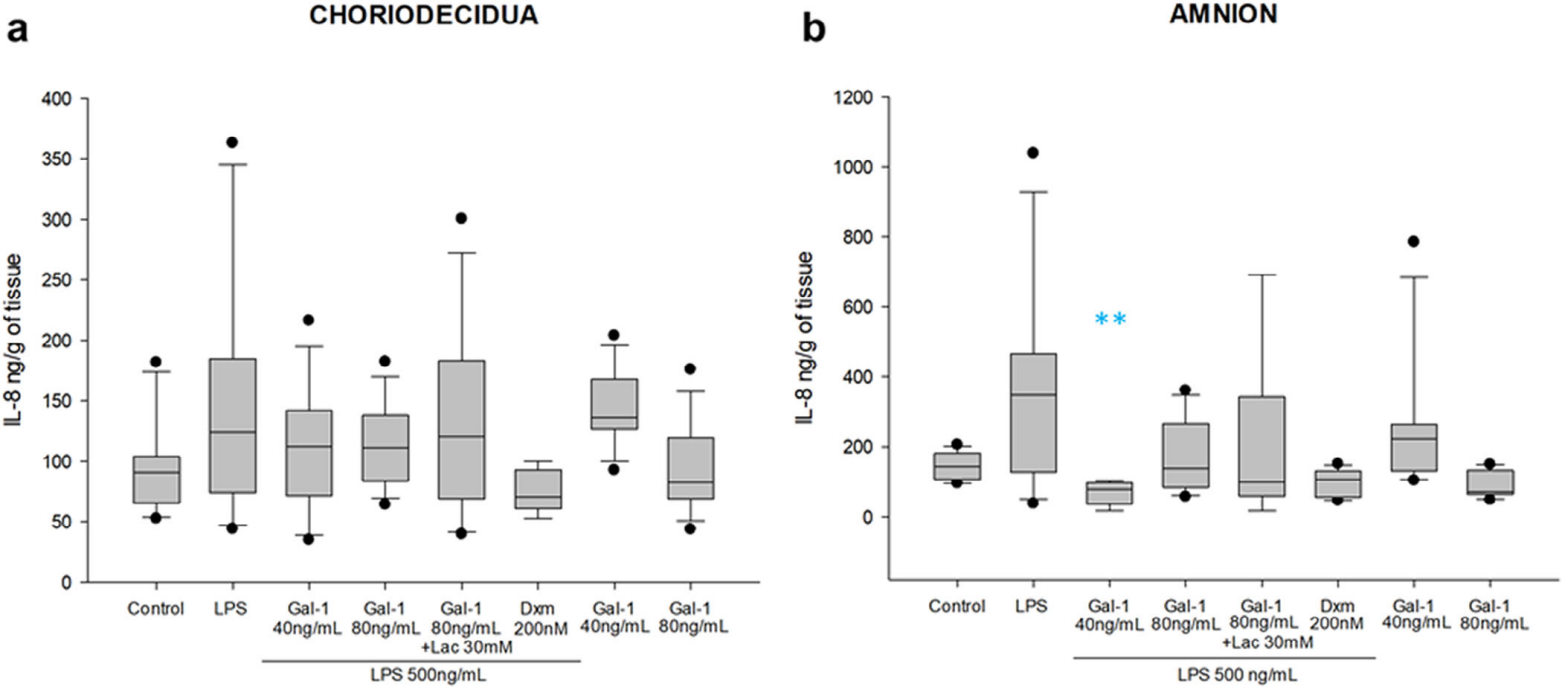
IL‐8 secretion profile in chorion and amnion. Quantification by ELISA in (a) chorion and (b) amnion culture medium. Plots are presented as boxes and whiskers: graphs show medians with interquartile range. LPS: lipopolysaccharide 500 ng/mL, Dxm: dexamethasone (200 nM), lac: Lac (30 mM). Significant differences versus *control, **LPS and ẟ versus Gal‐1 80ng+LPS (*p* ≤ 0.05) are indicated.

The amniotic stimulus with LPS increased the IL‐8 secretion by 1.36 times and 2.4 times in the chorion (124 [73.9–184.8] vs. 91 [65.3–104.1] ng/mL/g of tissue) and amnion (348.8 [127–465.1] vs. 143.5 [106.3–180.7] ng/mL/g of tissue), respectively. Gal‐1 did not statistically modulate IL‐8 in the chorion region. However, 40 ng of Gal‐1 significantly diminished IL‐8 secretion in the amnion compared to LPS treatment (77.5 [36.8–97.6] ng/mL/g of tissue). The treatment with Lac could not revert the anti‐IL‐8 effect of Gal‐1 in the amnion (99.5 [59.5–342.1] ng/mL/g of tissue). Although the Dxm reduced the level of IL‐8, this was not statistically significant, neither in choriodecidua nor amnion (Figure 7).

Overall, our results suggest that Gal‐1 reduces TNF‐α, IL‐6, IL‐1β, MCP‐1, RANTES‐ MIP‐1,α, and IL‐8 secretion in the human fetal membranes stimulated with LPS, mimicking an intraamniotic infection. Therefore, physiologic concentrations of Gal‐1 can act as an additional immunomodulator in the maternal–fetal interface.

### 3.3 Gal‐1 Downregulates the Inflammation‐Induced Secretion of MMP‐9

After this, we evaluated the effect of Gal‐1 on the MMP‐9 production by the chorioamniotic membranes inflamed by LPS. We studied their regulation in culture media and intra‐tissue of chorion and amnion regions.

**FIGURE 8:**
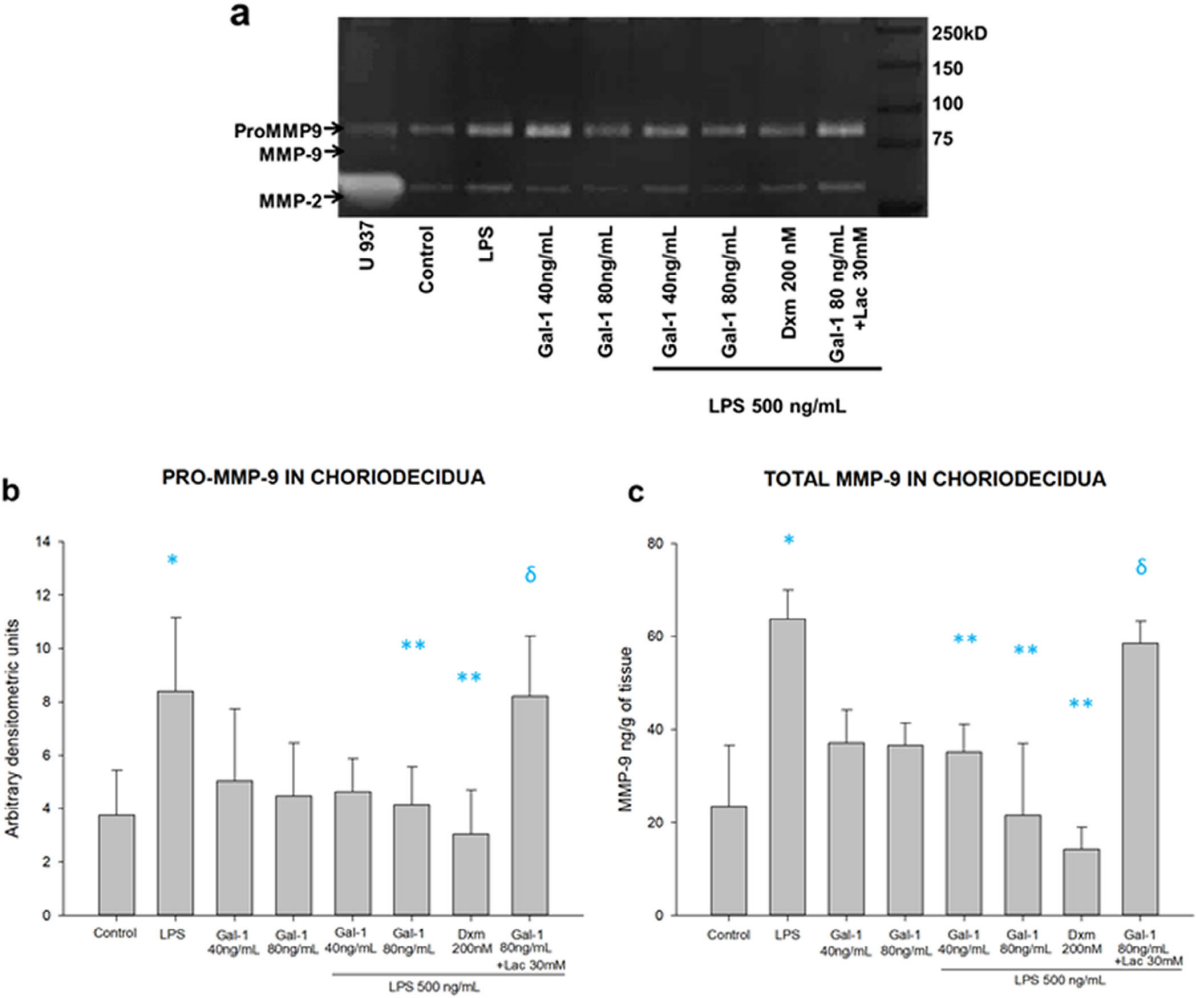
Activity and secretion profile of MMP‐9 in chorion. (a) Representative zymogram showing the enzymatic activity of pro MMP‐9 (lysis bands) in culture medium from the chorion. (b) Quantification by densitometric analysis of the zymogen. (c) Quantification of total MMP‐9 (active and proenzyme) by ELISA in chorion culture medium. LPS: lipopolysaccharide 500 ng/mL, Dxm: dexamethasone (200 nM), Lac (30 mM). Each bar represents the mean and standard deviation; significant differences (*p* ≤ 0.05) are indicated * versus control, ** versus LPS and ẟ versus Gal‐1 80ng+LPS.

In the chorion culture media, the amniotic stimulus with LPS was significantly induced by 2.2 times the pro‐MMP‐9 activity (8.3 vs. 3.7 arbitrary units). The pre‐treatment with 80 ng of Gal‐1 (4.1 arbitrary units) significantly reverted this induction in half. The treatment with Lac effectively blocks the anti‐MMP‐9 effect of galectin on pro‐MMP‐9 activity (8.2 arbitrary units) (Figures 8a,b). Similarly, the secreted total MMP‐9 in choriodecidua culture media shows a similar regulation pattern to that of the pro‐enzyme. LPS treatment induced 2.5 times MMP‐9 secretion in choriodecidua compared to basal levels (62 209 ± 7268 vs. 24 692 ± 11 920 pg/mL/g of tissue). Both doses of Gal‐1 inhibit MMP‐9 secretion by 40% and 48%, respectively (37 463 ± 6694 and 32 137 ± 9216 pg/mL/g of tissue), in comparison with LPS. Lac could revert the Gal‐1 downregulation of MMP‐9 secretion (57 788 ± 5476 pg/mL/g of tissue) (Figure 8c). The control with Lac reverted the effect of Gal‐1 in both regions of the membranes, and the co‐treatment with Dxm decreased the level of MMP‐9 in comparison with LPS alone.

**FIGURE 9:**
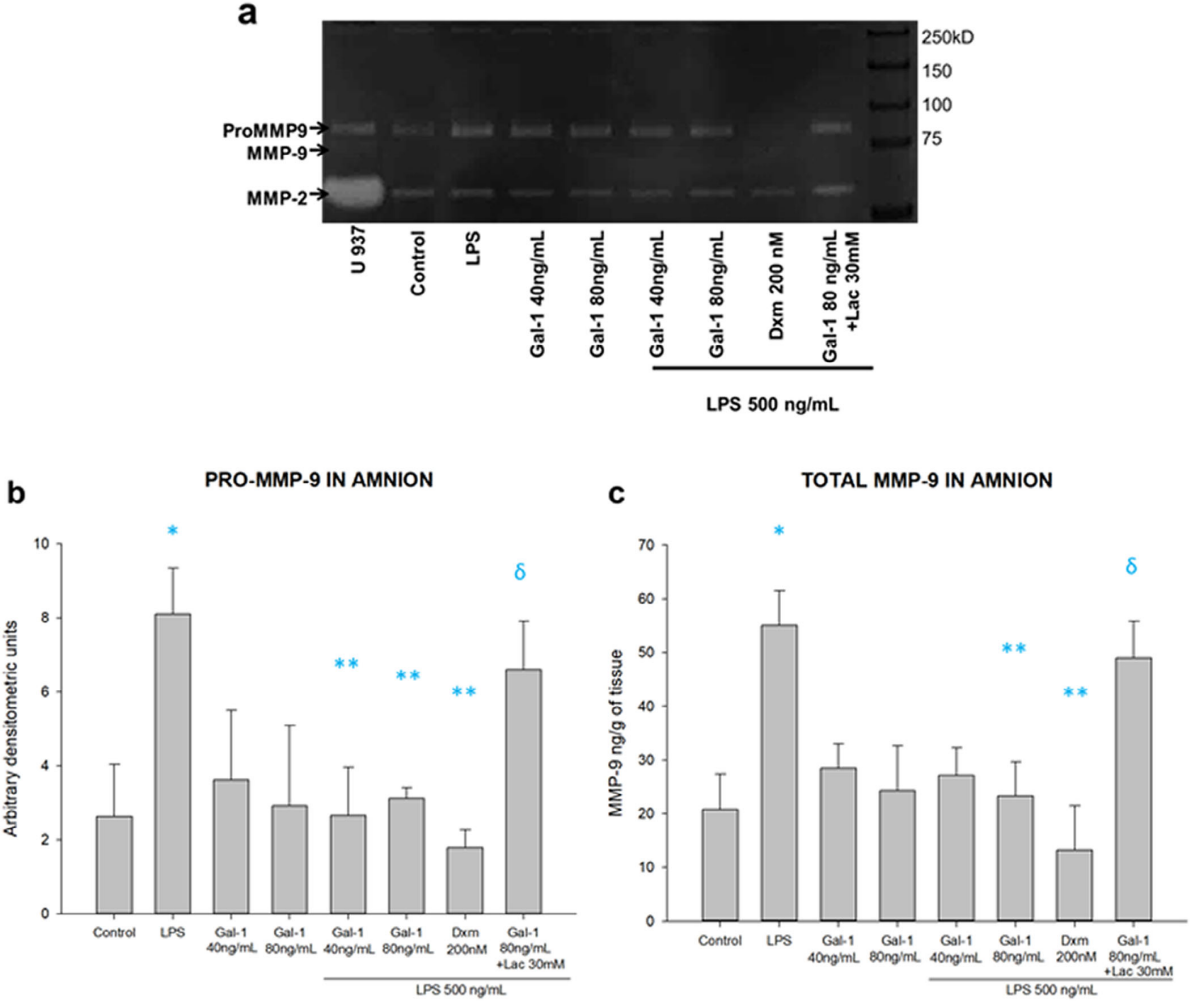
Activity and secretion profile of MMP‐9 in amnion. (a) Representative zymogram showing the enzymatic activity of pro MMP‐9 (lysis bands) in culture medium from the amnion. (b) Quantification by densitometric analysis of the zymogen. (c) Quantification of total MMP‐9 (active and proenzyme) by ELISA in amnion culture medium. LPS: lipopolysaccharide 500 ng/mL, Dxm: dexamethasone (200 nM), lac: Lac (30 mM). Each bar represents the mean and standard deviation; significant differences (*p* ≤ 0.05) are indicated * versus control, ** versus LPS and ẟ versus Gal‐1 80ng + LPS.

In the amnion culture media, pro‐MMP‐9 activity showed an upregulation by LPS in comparison to basal (8.0 vs. 2.7 arbitrary units). Both doses of Gal‐1 reduced pro‐MMP‐9 activity in comparison to LPS treatment (2.9 and 2.8 vs. 8.0 arbitrary units). Lac blocked the downregulation of pro‐MMP‐9 activity by Gal‐1 (6.2 arbitrary units) (Figure 9a,b). Likewise, secreted total (active and zymogen) MMP‐9 into amniotic culture media showed an upregulation by LPS treatment (52 789 ± 8708 vs. 21 476 ± 5940 pg/mL/g of tissue) and was significantly downregulated by 80 ng of Gal‐1 (24 579 ± 7995 pg/mL/g of tissue). This effect of Gal‐1 was effectively reversed by Lac (47 971 ± 7823 pg/mL/g of tissue) (Figure 9c). The control with Lac reverted the effect of Gal‐1 in both regions of the membranes, and the co‐treatment with Dxm decreased the level of MMP‐9 in comparison with LPS alone.

**FIGURE 10:**
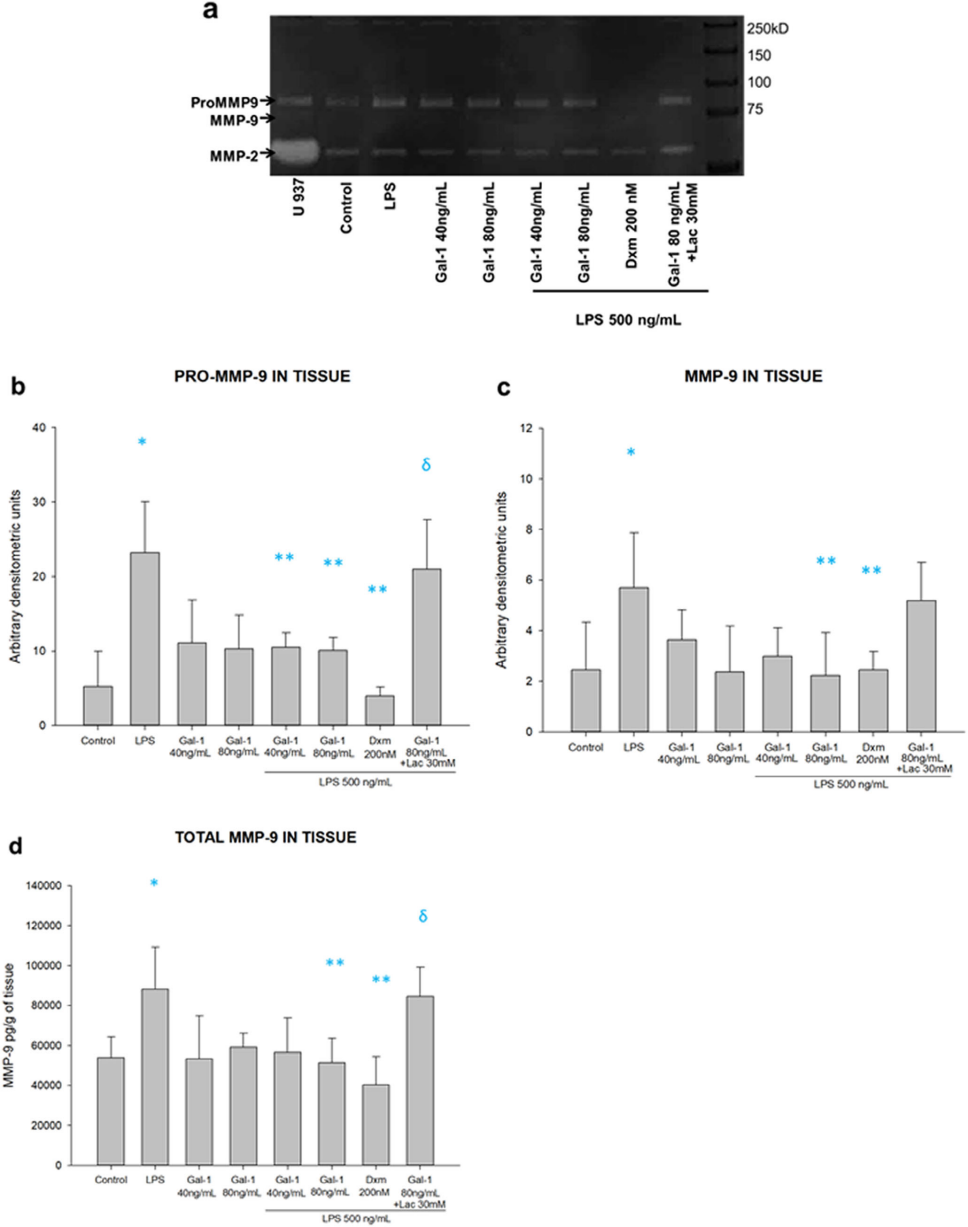
Activity and secretion profile of MMP‐9 in tissue. (a) Representative zymogram showing the enzymatic activity of Pro MMP‐9 (92 kDa) and the active isoform (82 kDa) in fetal membranes lysates. (b) Quantification by densitometric analysis of the zymogen and (c) the active form. (d) Quantification of total MMP‐9 (active and zymogenic) by ELISA. LPS: lipopolysaccharide 500 ng/mL, Dxm: dexamethasone (200 nM), lac: Lac (30 mM). Each bar represents the mean and standard deviation; significant differences versus * control, ** LPS, and ẟ versus Gal‐1 80ng+LPS (*p* ≤ 0.05) are indicated.

In addition, we measured intra‐tissue concentration of both pro‐MMP‐9 and total MMP‐9 in fused chorioamniotic membranes. LPS induced pro‐MMP‐9 tissular activity by four times in comparison to basal (24.6 ± 5.4 vs. 6.3 ± 3.6 arbitrary units). Pre‐treatment with both doses of Gal‐1 diminished the intra‐tissular pro‐MMP‐9 activity by 61% and 64%, respectively (9.5 ± 3.0 and 8.8 ± 2.9 arbitrary units). Lac reverted the anti‐MMP‐9 activity of Gal‐1 (22.2 ± 5.4 arbitrary units). Concerning the densitometric analysis of the active MMP‐9 band, LPS induced it by two times in comparison to basal (5.9 ± 1.9 vs. 3.0 ± 1.3 arbitrary units). Exposition to Gal‐1 at 80 ng diminished the intra‐tissular active MMP‐9 by 59% (2.4 ± 1.5 arbitrary units) compared to LPS stimulus. Lac also reverted active MMP‐9 downregulation by Gal‐1 (5.1 ± 1.6 arbitrary units). Finally, quantifying both active and zymogenic in intra‐tissue fetal membranes indicates the same pattern regulation of MMP‐9 by Gal‐1. LPS significantly induced total MMP‐9 tissular levels by 1.7 times compared to basal (92 558 ± 16 636 pg/mL/g of tissue). The addition of 80 ng of Gal‐1 significantly diminished total MMP‐9 intra‐tissue concentration by 42% (53 811 ± 9884 pg/mL/g of tissue) in comparison to LPS, and this effect was reversed by Lac (86 505 ± 12 719 pg/mL/g of tissue). Lac treatment reverted in a significant profile the effect of Gal‐1 in the pro‐MMP9 and total MMP‐9 in tissue. Dxm was effective in inhibiting the secretion of both isoforms of MMP‐9 in the tissue lysates (Figure 10).

### 3.4 Gal‐1 Protects the Structural Integrity of Fetal Membranes

**FIGURE 11:**
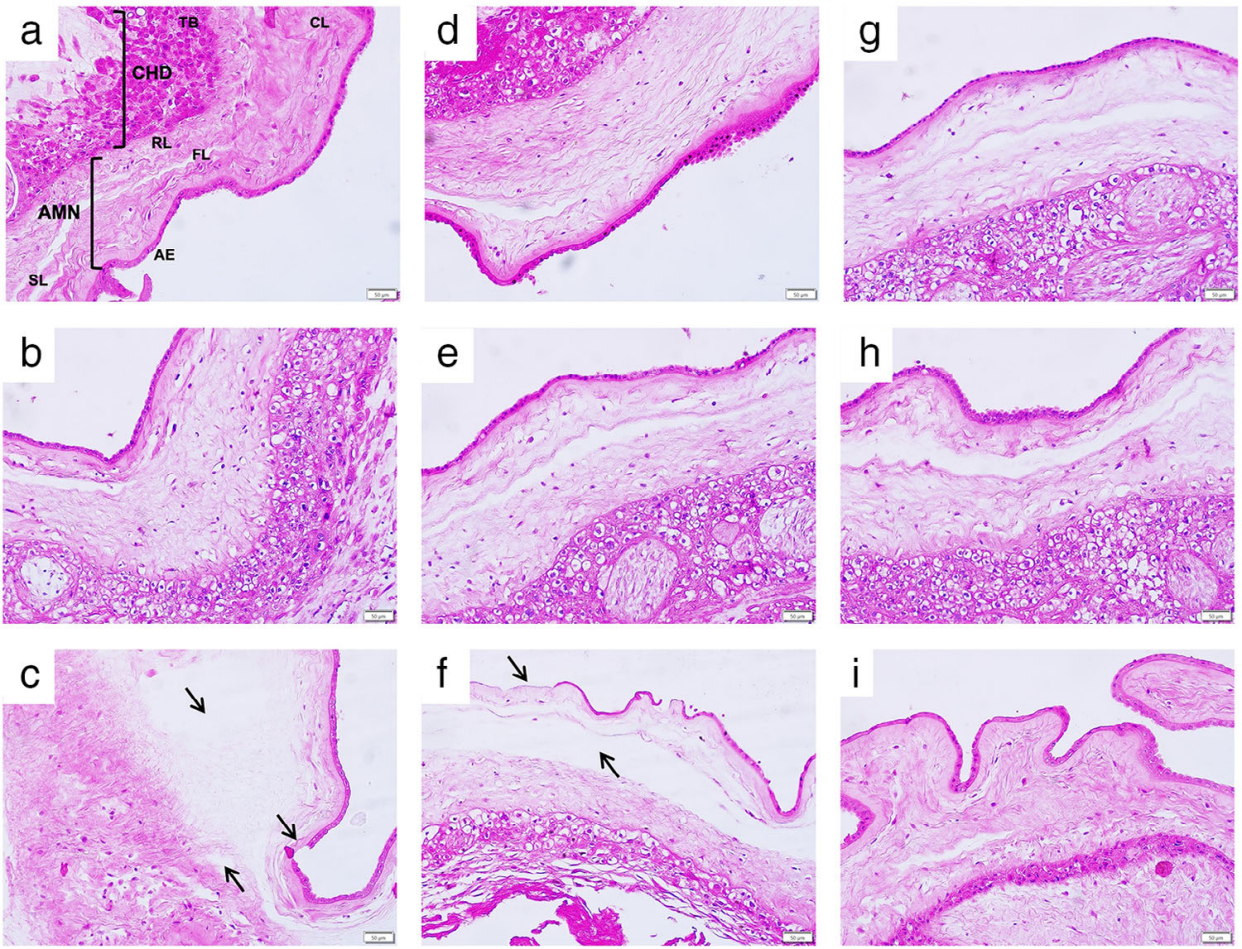
Efect of Gal‐1 treatment on structure of fetal membranes. Histological examination to evaluate structural changes damage to the fetal membranes after different treatments. (a) Control 0 h, (b) Control 72 h, (c) LPS (500 ng/mL) treatment, (d) co‐treatment with LPS (500 ng/mL) and Gal‐1 (40 ng/mL), (e) co‐treatment with LPS (500 ng/mL) and Gal‐1 (80 ng/mL), (f) co‐treatment with LPS (500 ng/mL) and Gal‐1 (80 ng/mL) and Lac (30 mM), (g) co‐treatment with LPS (500 ng/mL) and Dxm (200 nM), (h) Gal‐1 treatment(40 ng/mL), (i) Gal‐1 treatment(80 ng/mL). The amnion (AMN) of the fetal membranes is composed of amniotic epithelium (AE), fibrous layer (FL), spongy layer (SL), and reticular layer (RL), while the choriodecidua (CHD) contains trophoblast cells. Arrows show the zones with clear structural damage. Original magnification (20×).

Intending to evaluate the effect of Gal‐1 on the general structure of fetal membranes, we assess the histological characteristics of fetal membranes after the in vitro treatments. First, we confirm the absence of leukocytosis in the tissues. Under control conditions (0 and 72 h), the typical morphology of fetal membranes composed of amnion and choriodecidua was observed (Figure 11a,b). In contrast, after treatment with LPS, we observed an evident alteration in the connective tissue underneath the basement membrane of the amnion epithelium.

The fibrous, sponge, and reticular layers are swelling and structurally disorganized, causing an evident change in the thickness of the amniotic membrane. The collagen fibers encapsulating the trophoblasts in the choriodecidua are also disorganized. This significantly altered morphology is a characteristic of collagenolytic activity (Figure 11c). The tissue co‐treated with PRL and Gal‐1 40 ng/mL (Figure 11d) and Gal‐1 80 ng/mL (Figure 11e) showed minimal alteration in the connective tissue, suggesting the protective role of Gal‐1 over the tissue degradation. In the explants co‐treated with Lac, the structural architecture of the tissue looks altered mainly in the amnion region (Figure 11f). The Dxm protected the tissue from the damage associated with LPS (Figure 11g). The tissues co‐treated with Gal‐1 alone (Figure 11(h,i) maintain the normal morphology of fetal membranes.

## 4 Discussion

Successful pregnancy requires a precise time‐lapse equilibrium between immune tolerance and rejection mechanisms to permit the fetus to grow in the uterus as an immune‐privileged site. Fetal membranes are essential as a very selective immuno‐endocrine and physical barrier, maintaining the immune privilege of the amniotic cavity and maintaining structural stability and antimicrobial function, offering a homeostatic balance synchronizing signals between mother and fetus [44].

When an intraamniotic infection/inflammation occurs, this represents a colossal challenge for the maternal–fetal interface immunological privilege because the fetus will be “living” in a very hostile environment full of cytotoxic pro‐inflammatory cytokines. The fetal membranes are also exposed to inflammation modulators that are out of control and associated with chorioamnionitis and PPROM, which complicates 2%–3.5% of pregnancies and accounts for 30%–40% of PTBs [35, 45, 46]. Intraamniotic infection and inflammation are the primary causal underlying mechanisms of the disease in 25%–39% of the cases [35, 47–49].

Clinical evidence supporting the critical role of Gal‐1 during pregnancy indicates that alteration in the synthesis and secretion of Gal‐1 can be associated with multiple adverse outcomes. For example, in comparison with fertile women, patients with recurrent pregnancy loss showed considerably lower levels of circulating Gal‐1 and had a higher frequency of anti Gal‐1 auto‐Abs [50]. It has also been reported that Gal‐1, ‐3, and ‐9 levels in peripheral venous blood are elevated in preterm infants born in an inflammatory milieu such as amniotic infection syndrome or early‐onset sepsis. This supports the need to understand galectins’ potential immunomodulatory effects in preterm infants [51].

Herein, using an ex vivo model of human fetal membranes emulating an intraamniotic inflammation induced by LPS, we demonstrate that the pre‐stimulation of membranes with pregnancy physiological maternal serum concentrations of Gal‐1 [28] dampened, in a tissue‐specific profile, the secretion of IL‐1α, TNF‐α, and IL‐6, three pro‐inflammatory cytokines primarily and undebatable associated with the physiological and pathological onset of labor [44].

There is robust clinical and experimental evidence showing that inflammation derived from infection increases the risk of developing multiple adverse outcomes, including cerebral palsy and other neuropsychiatric conditions such as schizophrenia and autism [52]. Additionally, numerous animal models have shown that irrespective of the type of pathogen, and even in the absence of a pathogen, cytokine release is the link in producing fetal brain injury [53, 54, 55] and recurrent spontaneous miscarriage [56].

In the clinical context, there is evidence suggesting that compared with normal pregnancies, the Gal‐1 mRNA and protein levels are increased in the amniotic epithelium, chorioamniotic fibroblasts/myofibroblasts and macrophages, chorionic trophoblasts, and decidual stromal cells detected in amniotic epithelium of fetal membranes with chorioamnionitis; which has been interpreted as the evidence of key immunomodulatory role of Gal‐1 in human fetal membranes [35]. On the other hand, in comparison with healthy pregnant women and with PPROM without membrane infection, the mRNA of Gal‐1 increases in membranes with PPROM with histopathological confirmed inflammation [35]. Overexpression of Gal‐1 in fetal membranes might be linked to the weakening of their structure, increased susceptibility to infection, and, ultimately, their rupture. It has been proposed that Gal‐1 is an active barrier protecting the fetus against bacterial infection and facilitates phagocytosis of excessively producing maternal neutrophils [35].

As stated in previous lines, the regulation of inflammatory processes is of vital importance during pregnancy; in this sense, it is pertinent to highlight research such as that of Presicce et al., who using a model of intrauterine inflammation induced by LPS in rhesus macaques, demonstrated that TNF‐α signaling has a key role in modulating neutrophilic infiltration at the feto‐maternal interface. They suggested that blockade of TNF‐α signaling could be considered a therapeutic approach for intrauterine infection, the leading cause of PTB [57].

During an intrauterine infection, TNF‐α and IL‐1β play a central role in the defense mechanism; in the context of gestation, it has been demonstrated that intraamniotic infusion of IL‐1beta and TNF‐α, but not IL‐6 or IL‐8, can induce preterm labor in a model of pregnant rhesus monkeys [58]. This evidence supports the relevance of our findings reported herein, revealing that Gal‐1, in physiologic concentration during pregnancy, can inhibit the secretion of TNF‐alpha, IL‐1beta, and IL‐6 in a significant profile. These findings correlate with evidence indicating that the capacity of Gal‐1 to prevent fetal loss by mediating immune tolerance at the feto‐maternal interface suggests that Gal‐1 is one of the most upstream messengers in the cascade involved in sustaining successful pregnancy and supporting its capacity to skew the cytokine balance toward a TH2 profile [20].

The capacity of IL‐1β to onset a damage signal capable of provoking preterm labor was developed in a model of pregnant rhesus monkeys. Intraamniotic infusion of IL‐1beta rapidly produces intraamniotic TNF‐alpha, PGE2, and F2 alpha, followed by preterm uterine contractility. In this sterile inflammation, the neutrophils in the decidua are the significant source of TNF‐alpha and IL‐8 [59, 60]. The induction of preterm labor was also possible in mice that underwent intrauterine injection of recombinant human IL‐1β [61].

In our model, the human fetal membranes are responsive to LPS stimulus in the amnion region, secreting pro‐inflammatory cytokines; in this context, reports suggest that the fetal membrane inflammation is a process that involves the NLRP3 inflammasome, which once is activated through alarmins such as high mobility group box 1 (HMGB1) induces adverse fetal and neonatal outcomes [62, 63]. Upon activation, the inflammasome complex induces the autocatalytic cleavage of pro‐CASP1 into its active form, which can cleave pro‐IL‐1beta and pro‐IL‐18 into their mature and released forms with the consequent worsening of an inflammatory milieu [64].

Although there is no information about the effect of Gal‐1 upon the inflammasome in the maternal–fetal interface, an interesting finding indicates that Gal‐1 can ameliorate myocardial inflammation and oxidative damage in mice with myocarditis in a mechanism involving the inhibition of the NLRP3 inflammasome [65]. In fact, in an LPS‐challenged mice model designed to study acute lung injury, Gal‐1 alleviates inflammation and oxidative stress, suppressing the NLRP3 inflammasome too [66]. Considering these findings, it is our interest to plan an experimental approach to understand the role of Gal‐1 in activating inflammasome.

On the other hand, IL‐6 is an acknowledged inflammatory cytokine with a pleiotropic action. It mediates innate and adaptive immunity and multiple physiological processes from implantation to parturition, including protective and regenerative ones [67]. This cytokine is one of the best pro‐inflammatory biomarkers correlating with delivery timing, independent of the occurrence of intrauterine infection [68, 69]. Here, the pre‐treatment of fetal membranes with Gal‐1 downregulates the secretion of LPS‐induced IL‐6. This finding is central because increased levels of IL‐6 in the maternal–fetal interface could be detrimental to the tolerogenic environment in the uterus and compromise the pregnancy [70].

Previous evidence showed that in human decidual cells, Gal‐1 acts by inhibiting the stimulation of the LPS‐induced IκBζ expression, an NF‐κB regulator involved in IL‐6 gene transcription [71]. The immunological competencies of human fetal membranes during an infectious/inflammatory challenge are crucial to protect the fetus. In the context of intrauterine infection and chorioamnionitis, maternal immune activation may cause tissue injury and trigger maternal inflammatory/immune responses, releasing a plethora of effector molecules, with IL‐6 and IL‐8 having one of the key roles [72]. A vast number of studies have associated an increased level of IL‐6 in amniotic fluid and cervicovaginal lavage as an indicator of preterm labor and an increase in the risk of perinatal morbidity and mortality [73]. Additionally, clinical evidence indicates that compared with women having uneventful pregnancies, patients experiencing pregnancy loss exhibit increased IL‐6 concentration in plasma [74], serum [75], and PBMCs [76].

The exacerbation and perpetuation of inflammation in the maternal–fetal interface and its concomitant jeopardizing consequences are partially explained by the effect of multiple immune cells infiltrating the human fetal membranes [77]. The arrival of these cells results from the chemoattract effect of multiple chemokines, including IL‐8, RANTES, MCP1, and MIP1 alpha [78, 79]. Multiple models have mainly documented the secretion of these chemokines during pathological conditions such as preterm labor.

Our results indicate that membranes pre‐stimulated in vitro with human recombinant Gal‐1 in concentrations present during pregnancy could inhibit the secretion of MCP‐1, an integral chemotactic factor that recruits macrophages for the immune response [80]. Its production is important in normal and pathological pregnancies. In comparison with healthy pregnancies, clinical evidence suggests that the high level of MCP‐1 and MCP‐3 in amniotic fluid is significantly higher in preterm labor with chorioamnionitis, and their action is related to the degree of inflammatory infiltration in the placenta and fetal membranes [81, 82].

On the other hand, high concentrations MIP1‐α in the amniotic fluid have been associated with pathological scenarios such as preterm labor and the microbial invasion of the amniotic cavity (MIAC) [83]. This chemokine was produced by human fetal membranes in our model in response to LPS stimulation, and its secretion was downregulated significantly in those membranes previously stimulated with Gal‐1. This supports the anti‐inflammatory role of this lectin and confirms our original hypothesis.

Other chemokines, RANTES and IL‐8 are also closely related to chorioamnionitis damage mechanisms [84]. In the present study, the concentration of both chemokines increased in amnion and choriodecidua after LPS stimulation in the amniotic region.

Clinical evidence reveals that compared with controls, infants exposed to chorioamnionitis and admitted to the neonatal intensive care unit had higher levels of IL‐6, IL‐8, and RANTES in cord blood, which was associated with neurologic abnormalities [85]. In fact, a significant increase in RANTES concentration in early‐onset infections in neonates has been reported and proposed as a useful biomarker for diagnosing severe neonatal infection [86].

In our model, IL‐8 is only downregulated by Gal‐1 in the amnion region. This is a very interesting result because information suggests that IL‐8 dysregulation links perinatal systemic inflammation and atypical white matter development in preterm infants [87].

A large biomarker associated with chorioamnionitis, and premature rupture of membranes is the increase of MMP‐9, a gelatinase enzyme with the capacity to degrade different components of the robust extracellular matrix of fetal membranes. The increase of this enzyme is a positive predictive value for delivery timing prediction [68, 88].

In the present model, the pre‐treatment of Gal‐1 effectively and significantly decreased the concentration of the total (zymogen and active forms) of MMP‐9 secreted to the culture medium in response to the LPS stimulus and the forms present in the tissue lysate. This result demonstrates that Gal‐1 can effectively downregulate the inflammation and matrix degradation markers associated with preterm labor.

**FIGURE 12:**
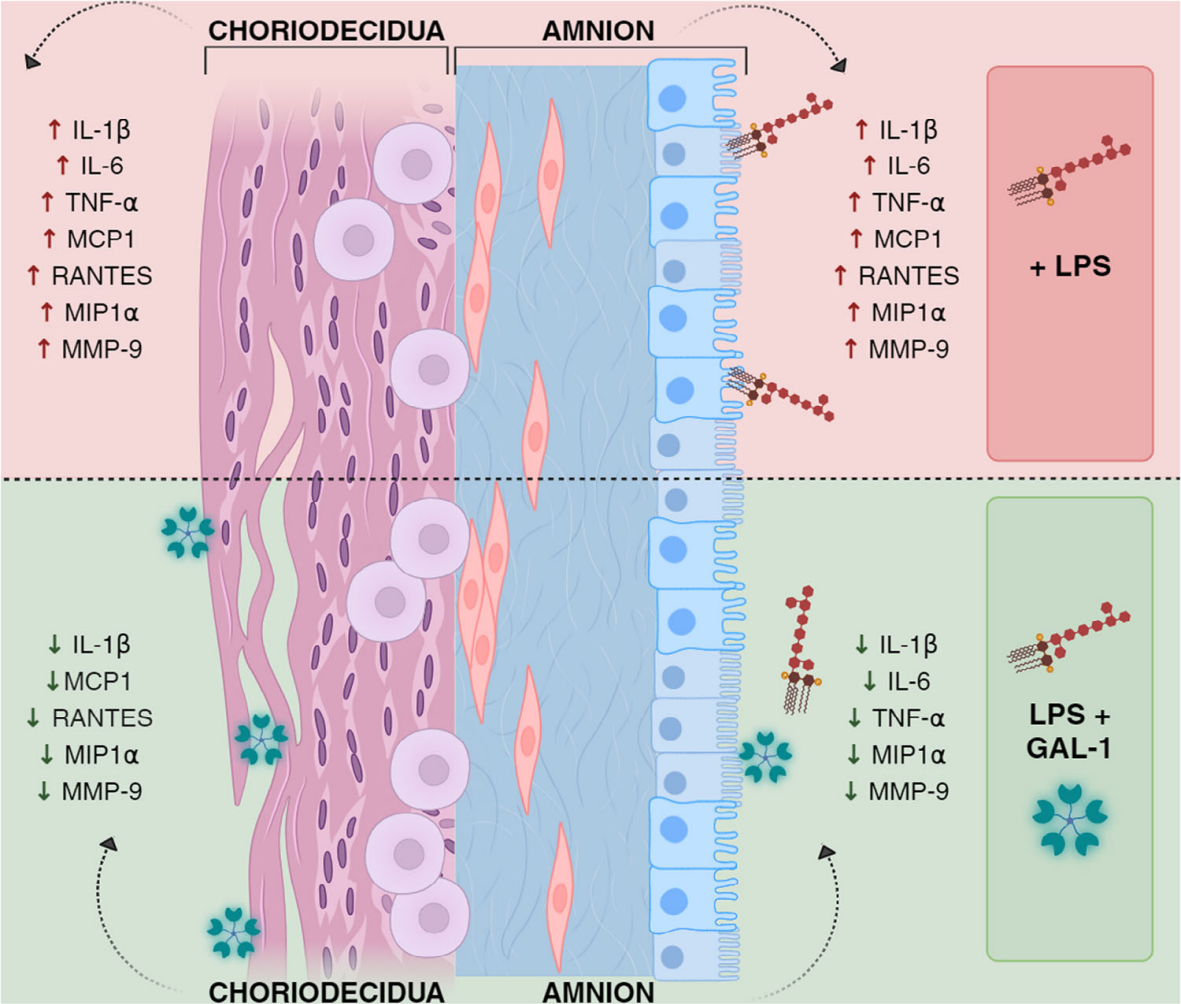
Role of Gal‐1 in the human fetal membranes. Gal‐1 induces a tissue‐specific anti‐inflammatory and anti‐degrative effect on human fetal membranes stimulated and inflamed with LPS. Amnion and choriodecidua regions respond differentially to the stimulus, with Gal‐1 offering a compartmentalized response to the fetus and mother.

The present work is the first to demonstrate that Gal‐1 can partially inhibit the inflammation process and the secretion and activity of MMP‐9, which is translated in a protective effect on the structural continuity of the human fetal membranes (Figure 12). However, although there is robust information about the role of fetal membranes in human labor, it is straightforward for us that future research should delve deeper into the mechanism of action of Gal‐1 in this tissue key in the maintenance of immune privilege [89].

## Ethics Statement

This study was approved by the Biosafety, Ethical and Research Committee from the Instituto Nacional de Perinatología (INPer)’’ and is registered under code number 2019‐1‐5. All methodological approaches were conducted according to the guidelines of the Declaration of Helsinki.

## Consent

Written informed consent was obtained voluntarily from all the mothers involved in the study, before cesarean section.

## Conflicts of Interest

The authors declare no conflicts of interest.

## Data Availability

The data used to support the findings of this study are available from the corresponding author upon request.

## References

[1] E. O. Ohuma , A. B. Moller , E. Bradley , et al., “National, Regional, and Global Estimates of Preterm Birth in 2020, With Trends From 2010: A Systematic Analysis,” Lancet 402, no. 10409 (2023): 1261–1271, 10.1016/S0140-6736(23)00878-4. 37805217

[2] J. Perin , A. Mulick , D. Yeung , et al., “Global, Regional, and National Causes of Under‐5 Mortality in 2000–19: An Updated Systematic Analysis With Implications for the Sustainable Development Goals,” Lancet Child Adolesc Health 6, no. 2 (2022): 106–115, 10.1016/S2352-4642(21)00311-4. 34800370 PMC8786667

[3] Secretaria de Salud . Cada Año Nacen En México 200 Mil Bebés Prematuros: Secretaría de Salud. 2022. Accessed July 29, 2024. https://www.gob.mx/salud/prensa/558‐cada‐ano‐nacen‐en‐mexico‐200‐mil‐bebes‐prematuros‐secretaria‐de‐salud?idiom=es#:~:text=Cada%20a%C3%B1o%20en%20M%C3%A9xico%20nacen,Perinatolog%C3%ADa%20(INPer)%20%E2%80%9CDr.

[4] R. Menon and L. S. Richardson , “Preterm Prelabor Rupture of the Membranes: A Disease of the Fetal Membranes,” Seminars in Perinatology 41, no. 7 (2017): 409–419, 10.1053/j.semperi.2017.07.012. 28807394 PMC5659934

[5] W. L. Lee , W. H. Chang , and P. H. Wang , “Risk Factors Associated with Preterm Premature Rupture of Membranes (PPROM),” Taiwanese Journal of Obstetrics and Gynecology 60, no. 5 (2021): 805–806, 10.1016/j.tjog.2021.07.004. 34507652

[6] R. Menon , L. S. Richardson , and M. Lappas , “Fetal Membrane Architecture, Aging and Inflammation in Pregnancy and Parturition,” Placenta 79 (2019): 40–45, 10.1016/j.placenta.2018.11.003. 30454905 PMC7041999

[7] E. Jung , R. Romero , L. Yeo , et al., “The Fetal Inflammatory Response Syndrome: The Origins of a Concept, Pathophysiology, Diagnosis, and Obstetrical Implications,” Seminars in Fetal & Neonatal Medicine 25, no. 4 (2020): 101146, 10.1016/j.siny.2020.101146. 33164775 PMC10580248

[8] J. Trowsdale and A. G. Betz , “Mother's Little Helpers: Mechanisms of Maternal‐fetal Tolerance,” Nature Immunology 7, no. 3 (2006): 241–246, 10.1038/ni1317. 16482172

[9] P. Terness , M. Kallikourdis , A. G. Betz , G. A. Rabinovich , S. Saito , and D. A. Clark , “Tolerance Signaling Molecules and Pregnancy: IDO, Galectins, and the Renaissance of Regulatory T Cells,” American Journal of Reproductive Immunology 58, no. 3 (2007): 238–254, 10.1111/j.1600-0897.2007.00510.x. 17681041

[10] N. G. Than , R. Romero , C. J. Kim , M. R. McGowen , Z. Papp , and D. E. Wildman , “Galectins: Guardians of Eutherian Pregnancy at the Maternal–Fetal Interface,” Trends in Endocrinology & Metabolism 23, no. 1 (2012): 23–31, 10.1016/j.tem.2011.09.003. 22036528 PMC3640805

[11] E. Menkhorst , N. G. Than , U. Jeschke , et al., “Medawar's PostEra: Galectins Emerged as Key Players During Fetal‐Maternal Glycoimmune Adaptation,” Frontiers in Immunology 12 (2021): 784473, 10.3389/fimmu.2021.784473. 34975875 PMC8715898

[12] M. Jovanović Krivokuća , A. Vilotić , M. Nacka‐Aleksić , et al., “Galectins in Early Pregnancy and Pregnancy‐Associated Pathologies,” International Journal of Molecular Sciences 23, no. 1 (2021): 69, 10.3390/ijms23010069. 35008499 PMC8744741

[13] F. A. van den Brûle , P. L. Fernandez , C. Buicu , et al., “Differential Expression of Galectin‐1 and Galectin‐3 during First Trimester human Embryogenesis,” Developmental Dynamics 209, no. 4 (1997): 399–405, 10.1002/(SICI)1097-0177(199708)209:4<;399::AID-AJA7>;3.0.CO;2-D. 9264263

[14] N. G. Than , R. Romero , A. Balogh , et al., “Galectins: Double‐Edged Swords in the Cross‐roads of Pregnancy Complications and Female Reproductive Tract Inflammation and Neoplasia,” Journal of Pathology and Translational Medicine 49, no. 3 (2015): 181–208, 10.4132/jptm.2015.02.25. 26018511 PMC4440931

[15] A. G. Blidner and G. A. Rabinovich , “Sweetening' Pregnancy: Galectins at the Fetomaternal Interface,” American Journal of Reproductive Immunology 69, no. 4 (2013): 369–382, 10.1111/aji.12090. 23406009

[16] S. H. Barondes , V. Castronovo , D. N. W. Cooper , et al., “Galectins: A Family of Animal β‐galactoside‐Binding Lectins,” Cell 76, no. 4 (1994): 597–598, 10.1016/0092-8674(94)90498-7. 8124704

[17] V. Sundblad , L. G. Morosi , J. R. Geffner , and G. A. Rabinovich , “Galectin‐1: A Jack‐of‐All‐Trades in the Resolution of Acute and Chronic Inflammation,” Journal of Immunology 199, no. 11 (2017): 3721–3730, 10.4049/jimmunol.1701172. 29158348

[18] G. Rabinovich , “Role of Galectins in Inflammatory and Immunomodulatory Processes,” Biochimica Et Biophysica Acta—General Subjects 1572, no. 2 (2002): 274–284, 10.1016/S0304-4165(02)00314-8. 12223275

[19] B. H. Bevan , D. C. Kilpatrick , W. A. Liston , J. Hirabayashi , and K. Kasai , “Immunohistochemical Localization of a Beta‐d‐Galactoside‐Binding Lectin at the Human Maternofetal Interface,” Histochemical Journal 26, no. 7 (1994): 582–586, 10.1007/BF00158592. 7960935

[20] S. M. Blois , J. M. Ilarregui , M. Tometten , et al., “A Pivotal Role for Galectin‐1 in Fetomaternal Tolerance,” Nature Medicine 13, no. 12 (2007): 1450–1457, 10.1038/nm1680. 18026113

[21] N. G. Than , R. Romero , O. Erez , et al., “Emergence of Hormonal and Redox Regulation of Galectin‐1 in Placental Mammals: Implication in Maternal–Fetal Immune Tolerance,” Proceedings of the National Academy of Sciences 105, no. 41 (2008): 15819–15824, 10.1073/pnas.0807606105. PMC2556362 18824694

[22] L. Vicovac , M. Jankovic , and M. Cuperlovic , “Galectin‐1 and ‐3 in Cells of the First Trimester Placental Bed,” Human Reproduction 13, no. 3 (1998): 730–735, 10.1093/humrep/13.3.730. 9572443

[23] E. Maquoi , F. A. van den Brûle , V. Castronovo , and J. M. Foidart , “Changes in the Distribution Pattern of Galectin‐1 and Galectin‐3 in human Placenta Correlates with the Differentiation Pathways of Trophoblasts,” Placenta 18, no. 5 (1997): 433–439, 10.1016/S0143-4004(97)80044-6. 9250706

[24] H. El‐Azzamy , A. Balogh , R. Romero , et al., “Characteristic Changes in Decidual Gene Expression Signature in Spontaneous Term Parturition,” Journal of Pathology and Translational Medicine 51, no. 3 (2017): 264–283, 10.4132/jptm.2016.12.20. 28226203 PMC5445200

[25] S. Borowski , N. Freitag , I. Urban , G. Michel , G. Barrientos , and S. M. Blois , “Examination of the Contributions of Maternal/Placental‐Derived Galectin‐1 to Pregnancy Outcome,” Methods in Molecular Biology 2442 (2022): 603–619, 10.1007/978-1-0716-2055-7_32. 35320548

[26] M. von Wolff , X. Wang , H. J. Gabius , and T. Strowitzki , “Galectin Fingerprinting in human Endometrium and Decidua during the Menstrual Cycle and in Early Gestation,” MHR: Basic Science of Reproductive Medicine 11, no. 3 (2004): 189–194, 10.1093/molehr/gah144. 15681515

[27] D. G. Boroń , A. Świetlicki , M. Potograbski , et al., “Galectin‐1 and Galectin‐9 Concentration in Maternal Serum: Implications in Pregnancies Complicated with Preterm Prelabor Rupture of Membranes,” Journal of Clinical Medicine 11, no. 21 (2022): 6330, 10.3390/jcm11216330. 36362558 PMC9658671

[28] I. Tirado‐Gonzalez , N. Freitag , G. Barrientos , et al., “Galectin‐1 Influences Trophoblast Immune Evasion and Emerges as a Predictive Factor for the Outcome of Pregnancy,” Molecular Human Reproduction 19, no. 1 (2013): 43–53, 10.1093/molehr/gas043. 23002109

[29] H. D. Kopcow , F. Rosetti , Y. Leung , D. S. J. Allan , J. L. Kutok , and J. L. Strominger , “T Cell Apoptosis at the Maternal–Fetal Interface in Early Human Pregnancy, Involvement of Galectin‐1,” Proceedings of the National Academy of Sciences 105, no. 47 (2008): 18472–18477, 10.1073/pnas.0809233105. PMC2587580 19011096

[30] M. Chen , J. L. Shi , Z. M. Zheng , Z. Lin , M. Q. Li , and J. Shao , “Galectins: Important Regulators in Normal and Pathologic Pregnancies,” International Journal of Molecular Sciences 23, no. 17 (2022): 10110, 10.3390/ijms231710110. 36077508 PMC9456357

[31] J. L. Chen , Y. Chen , D. X. Xu , and D. Z. Chen , “Possible Important Roles of Galectins in the Healing of Human Fetal Membranes,” Front Endocrinol 13 (2022): 941029, 10.3389/fendo.2022.941029. PMC9395672 36017312

[32] V. R. Aluvihare , M. Kallikourdis , and A. G. Betz , “Regulatory T Cells Mediate Maternal Tolerance to the Fetus,” Nature Immunology 5, no. 3 (2004): 266–271, 10.1038/ni1037. 14758358

[33] T. Tilburgs , D. L. Roelen , B. J. van der Mast , et al., “Evidence for a Selective Migration of Fetus‐Specific CD4+CD25 Bright Regulatory T Cells from the Peripheral Blood to the Decidua in Human Pregnancy,” The Journal of Immunology 180, no. 8 (2008): 5737–5745, 10.4049/jimmunol.180.8.5737. 18390759

[34] R. Shankar , M. P. Johnson , N. A. Williamson , et al., “Molecular Markers of Preterm Labor in the Choriodecidua,” Reproductive Sciences 17, no. 3 (2010): 297–310, 10.1177/1933719109353454. 20009011 PMC2852874

[35] N. G. Than , S. Kim , A. Abbas , et al., “Chorioamnionitis and Increased Galectin‐1 Expression in PPROM – An Anti‐Inflammatory Response in the Fetal Membranes?” American Journal of Reproductive Immunology 60, no. 4 (2008): 298–311, 10.1111/j.1600-0897.2008.00624.x. 18691335 PMC2784815

[36] D. G. Boron , J. Mikolajczyk‐Stecyna , A. Chmurzynska , G. Kurzawinska , W. Markwitz , and A. Seremak‐Mrozikiewicz , “Expression of Genes Encoding Galectin‐1 and Galectin‐9 in Placentas of Pregnancies With Preterm Prelabor Rupture of Membranes,” Ginekologia Polska Published online August 26, 2024, 10.5603/gpl.98834.

[37] B. Kaya , U. Turhan , S. Sezer , S. Kaya , İ. Dağ , and A. Tayyar , “Maternal Serum Galectin‐1 and Galectin‐3 Levels in Pregnancies Complicated with Preterm Prelabor Rupture of Membranes,” Journal of Maternal‐Fetal & Neonatal Medicine 33, no. 5 (2020): 861–868, 10.1080/14767058.2019.1637409. 31242786

[38] L. Dong , Q. Bai , W. Song , and C. Ban , “Abnormal Expression of Galectin‐1, ‐3 Leading to Unexplained Infertility by Decreasing Endometrial Receptivity: A Retrospective Analysis,” American Journal of Translational Research 15, no. 1 (2023): 493–501. 36777856 PMC9908466

[39] X. X. Jin , X. Ying , and M. Y. Dong , “Galectin‐1 Expression in the Serum and Placenta of Pregnant Women with Fetal Growth Restriction and Its Significance,” BMC Pregnancy Childbirth 21, no. 1 (2021): 14, 10.1186/s12884-020-03477-8. 33407212 PMC7789211

[40] V. Zaga , G. Estrada‐Gutierrez , J. Beltran‐Montoya , R. Maida‐Claros , R. Lopez‐Vancell , and F. Vadillo‐Ortega , “Secretions of Interleukin‐1β and Tumor Necrosis Factor α by Whole Fetal Membranes Depend on Initial Interactions of Amnion or Choriodecidua with Lipopolysaccharides or Group B Streptococci1,” Biology of Reproduction 71, no. 4 (2004): 1296–1302, 10.1095/biolreprod.104.028621. 15201199

[41] P. Flores‐Espinosa , I. Mancilla‐Herrera , A. Olmos‐Ortiz , L. Díaz , and V. Zaga‐Clavellina , “Culture of Human Fetal Membranes in a Two Independent Compartment Model: An Ex Vivo Approach,” Methods in Molecular Biology 2781 (2024): 61–69, 10.1007/978-1-0716-3746-3_6. 38502443

[42] I. V. Nesmelova , E. Ermakova , V. A. Daragan , et al., “Lactose Binding to Galectin‐1 Modulates Structural Dynamics, Increases Conformational Entropy, and Occurs with Apparent Negative Cooperativity,” Journal of Molecular Biology 397, no. 5 (2010): 1209–1230, 10.1016/j.jmb.2010.02.033. 20184898

[43] M. Massaro , A. J. Cagnoni , F. J. Medrano , et al., “Selective Modifications of Lactose and N‐acetyllactosamine With Sulfate and Aromatic Bulky Groups Unveil Unique Structural Insights in Galectin‐1‐Ligand Recognition,” Bioorganic & Medicinal Chemistry 94 (2023): 117480, 10.1016/j.bmc.2023.117480. 37774448

[44] R. Menon , “Fetal Inflammatory Response at the Fetomaternal Interface: A Requirement for Labor at Term and Preterm*,” Immunological Reviews 308, no. 1 (2022): 149–167, 10.1111/imr.13075. 35285967 PMC9188997

[45] T. F. McElrath , J. L. Hecht , O. Dammann , et al., “Pregnancy Disorders That Lead to Delivery Before the 28th Week of Gestation: An Epidemiologic Approach to Classification,” American Journal of Epidemiology 168, no. 9 (2008): 980–989, 10.1093/aje/kwn202. 18756014 PMC2720771

[46] L. Grönroos , P. Rautava , S. Setänen , et al., “Associations between the Aetiology of Preterm Birth and Mortality and Neurodevelopment up to 11 Years,” Acta Paediatrica 113, no. 3 (2024): 471–479, 10.1111/apa.17027. 37926858

[47] S. K. Srinivas and G. A. Macones , “Preterm Premature Rupture of the Fetal Membranes: Current Concepts,” Minerva Ginecologica 57, no. 4 (2005): 389–396. 16170284

[48] R. Romero , J. Espinoza , J. Kusanovic , et al., “The Preterm Parturition Syndrome,” British Journal of Obstetrics and Gynaecology 113, no. s3 (2006): 17–42, 10.1111/j.1471-0528.2006.01120.x. PMC7062298 17206962

[49] J. Santolaya‐Forgas , R. Romero , J. Espinoza , et al., “Prelabor Rupture of the Membranes,” in Clinical Obstetrics, eds. E. A. Reece and J. C. Hobbins (Hoboken, NJ: Wiley, 2007): 1130–1188, 10.1002/9780470753293.ch63.

[50] R. E. Ramhorst , L. Giribaldi , L. Fraccaroli , et al., “Galectin‐1 Confers Immune Privilege to Human Trophoblast: Implications in Recurrent Fetal Loss,” Glycobiology 22, no. 10 (2012): 1374–1386, 10.1093/glycob/cws104. 22752006

[51] K. Faust , N. Freitag , G. Barrientos , C. Hartel , and S. M. Blois , “Galectin‐Levels Are Elevated in Infants Born Preterm due to Amniotic Infection and Rapidly Decline in the Neonatal Period,” Frontiers in Immunology 11 (2021): 599104, 10.3389/fimmu.2020.599104. 33717050 PMC7949913

[52] U. Meyer , J. Feldon , M. Schedlowski , and B. K. Yee , “Immunological Stress at the Maternal–Foetal Interface: A Link between Neurodevelopment and Adult Psychopathology,” Brain, Behavior, and Immunity 20, no. 4 (2006): 378–388, 10.1016/j.bbi.2005.11.003. 16378711

[53] P. H. Patterson , “Immune Involvement in Schizophrenia and Autism: Etiology, Pathology and Animal Models,” Behavioural Brain Research 204, no. 2 (2009): 313–321, 10.1016/j.bbr.2008.12.016. 19136031

[54] M. J. Bell , J. M. Hallenbeck , and V. Gallo , “Determining the Fetal Inflammatory Response in an Experimental Model of Intrauterine Inflammation in Rats,” Pediatric Research 56, no. 4 (2004): 541–546, 10.1203/01.PDR.0000139407.89883.6B. 15295096

[55] Z. Cai , Z. L. Pan , Y. Pang , O. B. Evans , and P. G. Rhodes , “Cytokine Induction in Fetal Rat Brains and Brain Injury in Neonatal Rats After Maternal Lipopolysaccharide Administration,” Pediatric Research 47, no. 1 (2000): 64–64, 10.1203/00006450-200001000-00013. 10625084

[56] C. Zhang , X. Deng , X. Zhang , et al., “Association between Serum TNF‐α Levels and Recurrent Spontaneous Miscarriage: A Meta‐Analysis,” American Journal of Reproductive Immunology 75, no. 2 (2016): 86–93, 10.1111/aji.12447. 26585408

[57] P. Presicce , M. Cappelletti , P. Senthamaraikannan , et al., “TNF‐Signaling Modulates Neutrophil‐Mediated Immunity at the Feto‐Maternal Interface During LPS‐Induced Intrauterine Inflammation,” Frontiers in Immunology 11 (2020): 558, 10.3389/fimmu.2020.00558. 32308656 PMC7145904

[58] D. W. Sadowsky , K. M. Adams , M. G. Gravett , S. S. Witkin , and M. J. Novy , “Preterm Labor Is Induced by Intraamniotic Infusions of Interleukin‐1β and Tumor Necrosis Factor‐α but Not by Interleukin‐6 or Interleukin‐8 in a Nonhuman Primate Model,” American Journal of Obstetrics and Gynecology 195, no. 6 (2006): 1578–1589, 10.1016/j.ajog.2006.06.072. 17132473

[59] S. Baggia , M. G. Gravett , S. S. Witkin , G. J. Haluska , and M. J. Novy , “Interleukin‐Iβ Intra‐Amniotic Infusion Induces Tumor Necrosis Factor‐α, Prostaglandin Production, and Preterm Contractions in Pregnant Rhesus Monkeys,” Journal of the Society for Gynecologic Investigation 3, no. 3 (1996): 121–126, 10.1177/107155769600300304. 8796819

[60] P. Presicce , P. Senthamaraikannan , M. Alvarez , et al., “Neutrophil Recruitment and Activation in Decidua with Intra‐Amniotic IL‐1beta in the Preterm Rhesus Macaque1,” Biology of Reproduction 92, no. 2 (2015): 56, 10.1095/biolreprod.114.124420. 25537373 PMC4342792

[61] K. Yoshimura and E. Hirsch , “Effect of Stimulation and Antagonism of Interleukin‐1 Signaling on Preterm Delivery in Mice,” Journal of the Society for Gynecologic Investigation 12, no. 7 (2005): 533–538, 10.1016/j.jsgi.2005.06.006. 16202930

[62] J. Faro , R. Romero , G. Schwenkel , et al., “Intra‐amniotic Inflammation Induces Preterm Birth by Activating the NLRP3 Inflammasome†,” Biology of Reproduction 100, no. 5 (2019): 1290–1305, 10.1093/biolre/ioy261. 30590393 PMC6698670

[63] N. Gomez‐Lopez , R. Romero , V. Garcia‐Flores , et al., “Inhibition of the NLRP3 Inflammasome Can Prevent Sterile Intra‐Amniotic Inflammation, Preterm Labor/Birth, and Adverse Neonatal Outcomes†,” Biology of Reproduction 100, no. 5 (2019): 1306–1318, 10.1093/biolre/ioy264. 30596885 PMC6497524

[64] J. Fu and H. Wu , “Structural Mechanisms of NLRP3 Inflammasome Assembly and Activation,” Annual Review of Immunology 41 (2023): 301–316, 10.1146/annurev-immunol-081022-021207. PMC10159982 36750315

[65] L. Shen , K. Lu , Z. Chen , Y. Zhu , C. Zhang , and L. Zhang , “Pre‐Treatment with Galectin‐1 Attenuates Lipopolysaccharide‐Induced Myocarditis by Regulating the Nrf2 Pathway,” European Journal of Histochemistry 67, no. 4 (2023): 3816, 10.4081/ejh.2023.3816. 38058290 PMC10773196

[66] X. T. Huang , W. Liu , Y. Zhou , et al., “Galectin‐1 Ameliorates Lipopolysaccharide‐Induced Acute Lung Injury via AMPK‐Nrf2 Pathway in Mice,” Free Radical Biology and Medicine 146 (2020): 222–233, 10.1016/j.freeradbiomed.2019.11.011. 31711983

[67] A. Vilotić , M. Nacka‐Aleksić , A. Pirković , Ž Bojić‐Trbojević , D. Dekanski , and M. Jovanović Krivokuća , “IL‐6 and IL‐8: An Overview of Their Roles in Healthy and Pathological Pregnancies,” International Journal of Molecular Sciences 23, no. 23 (2022): 14574, 10.3390/ijms232314574. 36498901 PMC9738067

[68] S. Feduniw , M. Pruc , M. Ciebiera , et al., “Biomarkers for Pregnancy Latency Prediction After Preterm Premature Rupture of Membranes–A Systematic Review,” International Journal of Molecular Sciences 24, no. 9 (2023): 8027, 10.3390/ijms24098027. 37175733 PMC10178250

[69] P. Presicce , C. Roland , P. Senthamaraikannan , et al., “IL‐1 and TNF Mediates IL‐6 Signaling at the Maternal‐Fetal Interface During Intrauterine Inflammation,” Frontiers in Immunology 15 (2024): 1416162, 10.3389/fimmu.2024.1416162. 38895127 PMC11183269

[70] T. Laisk , A. L. G. Soares , T. Ferreira , et al., “The Genetic Architecture of Sporadic and Multiple Consecutive Miscarriage,” Nature Communications 11, no. 1 (2020): 5980, 10.1038/s41467-020-19742-5. PMC7689465 33239672

[71] F. Gómez‐Chávez , V. Castro‐Leyva , A. Espejel‐Núñez , et al., “Galectin‐1 Reduced the Effect of LPS on the IL‐6 Production in Decidual Cells by Inhibiting LPS on the Stimulation of IκBζ,” Journal of Reproductive Immunology 112 (2015): 46–52, 10.1016/j.jri.2015.07.002. 26226212

[72] E. Gruys , M. J. M. Toussaint , T. A. Niewold , and S. J. Koopmans , “Acute Phase Reaction and Acute Phase Proteins,” Journal of Zhejiang University‐SCIENCE B 6, no. 11 (2005): 1045–1056, 10.1631/jzus.2005.B1045. 16252337 PMC1390650

[73] A. Leaños‐Miranda , A. G. Nolasco‐Leaños , R. I. Carrillo‐Juárez , C. J. Molina‐Pérez , I. Isordia‐Salas , and K. L. Ramírez‐Valenzuela , “Interleukin‐6 in Amniotic Fluid: A Reliable Marker for Adverse Outcomes in Women in Preterm Labor and Intact Membranes,” Fetal Diagnosis and Therapy 48, no. 4 (2021): 313–320, 10.1159/000514898. 33794521

[74] J. Calleja‐Agius , E. Jauniaux , A. R. Pizzey , and S. Muttukrishna , “Investigation of Systemic Inflammatory Response in First Trimester Pregnancy Failure,” Human Reproduction 27, no. 2 (2012): 349–357, 10.1093/humrep/der402. 22131390

[75] R. Thaker , H. Oza , V. Verma , M. Gor , and S. Kumar , “The Association of Circulatory Cytokines (IL‐6 and IL‐10) Level With Spontaneous Abortion–A Preliminary Observation,” Reproductive Sciences 28, no. 3 (2021): 857–864, 10.1007/s43032-020-00292-6. 32789572

[76] L. Zhao , L. Han , G. Hei , et al., “Diminished miR‐374c‐5p Negatively Regulates IL (Interleukin)‐6 in Unexplained Recurrent Spontaneous Abortion,” Journal of Molecular Medicine 100, no. 7 (2022): 1043–1056, 10.1007/s00109-022-02178-3. 35689099

[77] C. Zhang , J. Cao , M. Xu , D. Wu , W. Li , and Y. Chang , “The Role of Neutrophils in Chorioamnionitis,” Frontiers in Immunology 14 (2023): 1198831, 10.3389/fimmu.2023.1198831. 37475854 PMC10354368

[78] V. Khandre , J. Potdar , and A. Keerti , “Preterm Birth: An Overview,” Cureus 14, no. 12 (2022): e33006, 10.7759/cureus.33006. 36712773 PMC9879350

[79] C. J. Kim , R. Romero , P. Chaemsaithong , N. Chaiyasit , B. H. Yoon , and Y. M. Kim , “Acute Chorioamnionitis and Funisitis: Definition, Pathologic Features, and Clinical Significance,” American Journal of Obstetrics and Gynecology 213, no. 4 (2015): S29–S52, 10.1016/j.ajog.2015.08.040. 26428501 PMC4774647

[80] Z. Lin , J. L. Shi , M. Chen , Z. M. Zheng , M. Q. Li , and J. Shao , “CCL2: An Important Cytokine in Normal and Pathological Pregnancies: A Review,” Frontiers in Immunology 13 (2023): 1053457, 10.3389/fimmu.2022.1053457. 36685497 PMC9852914

[81] R. M. Holst , R. Laurini , B. Jacobsson , et al., “Expression of Cytokines and Chemokines in Cervical and Amniotic Fluid: Relationship to Histological Chorioamnionitis,” Journal of Maternal‐Fetal & Neonatal Medicine 20, no. 12 (2007): 885–893, 10.1080/14767050701752601. 18050018

[82] M. S. Esplin , R. Romero , T. Chaiworapongsa , et al., “Monocyte Chemotactic Protein‐1 Is Increased in the Amniotic Fluid of Women Who Deliver Preterm in the Presence or Absence of Intra‐Amniotic Infection,” Journal of Maternal‐Fetal & Neonatal Medicine 17, no. 6 (2005): 365–373, 10.1080/14767050500141329. 16009638

[83] R. Romero , R. Gomez , M. Galasso , et al., “Macrophage Inflammatory Protein‐1α in Term and Preterm Parturtition: Effect of Microbial Invasion of the Amniotic Cavity,” American Journal of Reproductive Immunology 32, no. 2 (1994): 108–113, 10.1111/j.1600-0897.1994.tb01101.x. 7826499

[84] G. Sullivan , P. Galdi , N. Borbye‐Lorenzen , et al., “Preterm Birth Is Associated With Immune Dysregulation Which Persists in Infants Exposed to Histologic Chorioamnionitis,” Frontiers in Immunology 12 (2021): 722489, 10.3389/fimmu.2021.722489. 34512648 PMC8430209

[85] L. F. Shalak , A. R. Laptook , H. S. Jafri , O. Ramilo , and J. M. Perlman , “Clinical Chorioamnionitis, Elevated Cytokines, and Brain Injury in Term Infants,” Pediatrics 110, no. 4 (2002): 673–680, 10.1542/peds.110.4.673. 12359779

[86] M. Stojewska , M. Wąsek‐Buko , B. Jakub , et al., “Evaluation of Serum Chemokine RANTES Concentration as a Biomarker in the Diagnosis of Early‐Onset Severe Infections in Neonates,” Postepy Higieny I Medycyny Doswiadczalnej 70 (2016): 272–279, 10.5604/17322693.1198990. 27117103

[87] G. Sullivan , P. Galdi , M. B. Cabez , et al., “Interleukin‐8 Dysregulation Is Implicated in Brain Dysmaturation Following Preterm Birth,” Brain, Behavior, and Immunity 90 (2020): 311–318, 10.1016/j.bbi.2020.09.007. 32920182

[88] H. Flores‐Herrera , G. García‐López , N. F. Díaz , et al., “An Experimental Mixed Bacterial Infection Induced Differential Secretion of Pro‐inflammatory Cytokines (IL‐1β, TNFα) and proMMP‐9 in Human Fetal Membranes,” Placenta 33, no. 4 (2012): 271–277, 10.1016/j.placenta.2012.01.007. 22280559

[89] R. Menon , F. Behnia , J. Polettini , and L. S. Richardson , “Novel Pathways of Inflammation in Human Fetal Membranes Associated With Preterm Birth and Preterm Pre‐Labor Rupture of the Membranes,” Seminars in Immunopathology 42, no. 4 (2020): 431–450, 10.1007/s00281-020-00808-x. 32785751 PMC9296260

